# ZrO_2_ Aerogel‐Supported Pd Nanoparticles for Photothermal CO_2_ Reduction

**DOI:** 10.1002/advs.76221

**Published:** 2026-06-25

**Authors:** David Kiwic, Linard Räz, Elena Tervoort, Markus Niederberger

**Affiliations:** ^1^ Laboratory For Multifunctional Materials Department of Materials ETH Zurich Zurich Switzerland

**Keywords:** aerogel, CO_2_ reduction, palladium, photothermal catalysis, reverse water‐gas shift, zirconia

## Abstract

Decarbonizing chemical processes requires sustainable heat sources that can replace fossil‐derived fuels and feedstocks. In photothermal catalysis, heat is supplied by converting light directly on the catalyst surface. This direct heating eliminates the need for external heat sources and exchangers and enables efficient use of solar or LED light sources. Aerogel‐supported catalysts are particularly effective for photothermal applications due to their combination of transparency, low thermal conductivity, and high surface area, which allow deep light penetration and minimal heat loss. Using a scalable wet‐impregnation approach, palladium (Pd) was deposited on aerogel granules composed of zirconia (ZrO_2_) nanocrystals. The nanocrystals were gelled and shaped into spherical granules. Negatively charged palladium‐ethylenediaminetetraacetic acid (Pd‐EDTA) complexes were adsorbed onto the positively charged gel surface, followed by supercritical drying, calcination, and reduction under hydrogen to form well‐dispersed Pd nanoparticles. Under 4.8 W cm^−2^ concentrated white LED illumination, the Pd/ZrO_2_ aerogel reached temperatures up to 300°C, driving carbon dioxide (CO_2_) reduction to carbon monoxide (CO) with ∼96% selectivity. Co‐deposition of indium (In) stabilized the catalytic activity and increased CO selectivity above 99%. Compared to powdered catalysts of the same composition, the PdIn/ZrO_2_ aerogel spheres produced ∼four times more CO, highlighting the combined benefit of efficient light absorption and low thermal conductivity.

## Graphical abstract

Nanoparticle‐based aerogels are promising catalysts for photothermal reactions due to optical transparency, high surface area, and low thermal conductivity. Here, we present deposition of Pd nanoparticles on ZrO_2_ aerogel granules by a wet‐impregnation approach. Under LED illumination, the aerogel reached 300°C, driving CO_2_ reduction to CO with ∼96% selectivity. Co‐deposition of indium improved stability, selectivity, and CO production.

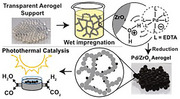

## 1 Introduction

The chemical industry is among the largest industrial sources of CO_2_ emissions, alongside cement, aviation, and steel. This is primarily due to two factors: the heat required for chemical transformations is almost entirely supplied by fossil fuels, and carbon‐containing feedstocks are also fossil‐derived. These contribute roughly 1 Gt of annual CO_2_ each, together accounting for about 5% of global emissions [1].

Most chemical processes require either low (<150°C) or medium‐temperature heat (150°C–400°C) [2]. For low‐temperature heat, techno‐economic analyses identify heat pumps powered by renewable electricity as the most cost‐effective decarbonization option [3, 4]. Medium‐temperature heat remains difficult to decarbonize due to lower technology readiness and uncertain costs [5]. Promising strategies include resistive electrical heating, combustion of renewable gases, and solar thermal systems. Electrical heating and hydrogen offer flexibility because they can be powered by various renewable sources. However, when solar power is the primary renewable resource, converting sunlight to electricity and then to heat incurs substantial losses, with total light‐to‐heat efficiencies often below 20% for resistive heating and 15% for electrolytic hydrogen [6]. In contrast, concentrated solar thermal systems convert sunlight directly into heat and can reach efficiencies up to 60% [7], making them particularly promising for chemical industries in regions with high solar irradiance.

Integrating solar thermal heat into a chemical plant typically involves concentrating sunlight with mirrors onto a collector tube filled with a thermal fluid such as steam or oil, which then transfers heat to a separate reactor containing the catalyst [8]. Emerging designs replace the collector tube with a glass tube packed with the catalyst, exposing the reactor bed directly to concentrated sunlight [9]. In this configuration, the catalyst itself converts light into reaction‐driving heat, a concept known as photothermal catalysis [10, 11, 12]. By eliminating the intermediate heat‐transfer step, this approach reduces thermal losses and simplifies system design by removing the need for a separate heat‐transfer loop.

In principle, conventional metal oxide‐supported catalysts can be repurposed for photothermal applications [13, 14, 15, 16, 17]. However, realizing the full potential of photothermal catalysis requires careful catalyst design to achieve both high chemical activity and efficient light‐to‐heat conversion. Plasmonic metal nanoparticles (Au, Ag, and Cu) are often employed because they strongly absorb light and generate highly localized heat [17, 18, 19]. To overcome the limited intrinsic reactivity of plasmonic metals, recent studies have introduced antenna‐reactor architectures, pairing plasmonic light absorbers with catalytically active metals to enhance photothermal conversion [20, 21, 22, 23]. Many photothermal catalysts in the literature are fabricated using substrate‐based methods such as colloidal lithography, self‐assembly, or physical vapor deposition. These nanostructured model architectures are typically highly regular, allowing precise control over particle size, spacing, and composition, which enables detailed mechanistic investigations [24, 25, 26, 27]. While ideal for studying structure‐activity relationships, the total catalyst volume is often very small, limiting overall product formation even when intrinsic reaction rates are high. In more practical powder‐supported catalysts light is strongly scattered restricting penetration depths to only tens of micrometers [22]. Thus, most photothermal catalysts suffer from limited heated volumes. Overcoming this constraint requires the development of support materials that retain the scalability of powders while permitting efficient light penetration into the catalyst materials.

Transparent aerogels combine minimal light scattering with high surface area and open porosity, making them attractive for light‐driven catalysis. Metal nanoparticles have previously been incorporated into aerogels by co‐gelation, yielding aerogel‐supported catalysts with well‐distributed catalytically active nanoparticles [28, 29, 30, 31]. While such materials have mostly been investigated for photocatalysis, their potential for photothermal catalysis is considerable because aerogels also possess exceptionally low thermal conductivity. When light penetrates the aerogel and is absorbed by metallic nanoparticles, the resulting heat is retained more effectively than in conventional supports, minimizing losses to the surroundings. However, co‐gelation approaches require the cumbersome pre‐synthesis of metal nanoparticles and precise control of their dispersibility during gelation, which can be difficult to scale. Wet‐impregnation strategies offer a scalable alternative, but they often compromise transparency or disrupt the delicate pore architecture of the aerogel [32, 33]. Impregnation of wet gels before drying can preserve gel transparency yet has largely relied on metal‐precursors soluble in organic solvents [34, 35]. Such precursors are often costly and only available for a small number of metals, limiting compositional flexibility in catalyst design.

Here, we present a scalable aqueous strategy for fabricating translucent, aerogel‐supported photothermal catalysts. Zirconia nanocrystals are gelled into uniform, translucent spheres of ∼1 mm diameter and impregnated with water‐soluble palladium‐ethylenediaminetetraacetic acid (Pd‐EDTA) complexes. The EDTA ligand forms a negatively charged palladium complex that adsorbs efficiently onto the positively charged ZrO_2_ surface, offering a versatile route to incorporate a wide range of catalytically active metals [36, 37, 38, 39]. Supercritical drying preserves both translucency and porosity, while subsequent calcination removes organics cleanly. In situ reduction then generates highly dispersed, strongly light‐absorbing Pd nanoparticles throughout the aerogel network. The resulting Pd/ZrO_2_ aerogel spheres combine efficient light absorption, low thermal conductivity, and high surface area, enabling optimal photothermal CO_2_ reduction. Co‐deposition of indium further enhances catalyst stability and improves CO selectivity. By coupling solar‐driven heating with CO_2_ conversion, these aerogel catalysts address two central decarbonization challenges for the chemical industry: supplying renewable process heat and displacing fossil‐derived carbon feedstocks.

## 2 Results and Discussion

For an efficient catalytic system the catalyst's activity must be balanced with its mass transport properties. When mass transport is limiting, improvements in catalytic activity do not translate into higher yields. For aerogel‐based catalysts, transport was shown to be only diffusional due to their mesoporous structure [40]. In a recent study, we showed that transport limitations can be mitigated by reducing the bulk size of the aerogel. Instead of casting large monoliths, wet gels were shaped into worm‐like granules, which outperformed monoliths in photocatalytic methanol combustion [41]. Although these elongated granules facilitate scalable processing, aerogel spheres offer superior mechanical properties and more uniform bed packing in the photoreactor. Uniform packing ensures a compact reactor with predictable gas flow, minimizes dead space between granules, and helps avoid local hot or cold spots that could compromise catalyst performance.

**FIGURE 1:**
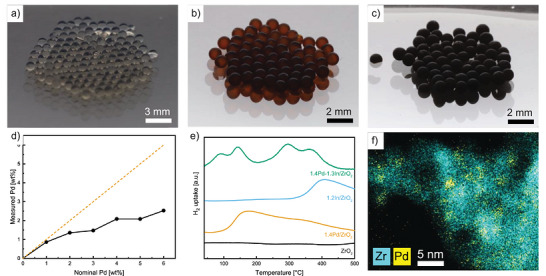
Metal‐loading of spherical ZrO_2_ aerogel granules for photothermal catalysis. (a) Photograph of transparent ZrO_2_ aerogel spheres. (b) Translucent brown ZrO_2_ spheres after wet impregnation with Pd‐EDTA complexes, and calcination at 300°C. (c) Black ZrO_2_ spheres after reduction with H_2_ forming Pd^0^, indicating strong visible‐light absorption. (d) XRF analysis of calcined Pd/ZrO_2_ aerogels showing measured Pd contents relative to the nominal Pd amounts introduced during impregnation (orange dashed line). (e) H_2_‐TPR profiles showing reducibility of bare ZrO_2_, monometallic In/ZrO_2_ and Pd/ZrO_2_, and bimetallic PdIn/ZrO_2_ catalysts. (f) STEM‐EDX map of reduced Pd/ZrO_2_ depicting well‐dispersed Pd nanoparticles on the ZrO_2_ surface with an average diameter of ∼1.5 nm.

To produce highly regular gel spheres, we employed the method outlined in our previous study [41]. ZrO_2_ nanocrystals (∼3 nm) were synthesized by a nonaqueous sol‐gel route, washed, and redispersed in water, where their positive surface charge at low pH yields a highly stable colloidal dispersion. Gelation was then induced by the addition of the non‐solvent 1,4‐dioxane, as explained in detail elsewhere [42]. To obtain spherical granules, the partially gelled dispersion (“pre‐gel”) was loaded into a syringe and extruded dropwise into a two‐phase system consisting of a silicone‐oil layer above a dioxane/water mixture heated to 60°C. Upon contact with the silicone oil, the droplets formed uniform spheres under surface tension and rapidly solidified during their descent before crossing into the solvent‐rich lower layer, where gelation was completed. During extrusion, the syringe containing the pre‐gel was cooled with ice water to suppress premature gelation and ensure consistent sphere formation. After gelation, the silicone oil was removed, and the ZrO_2_ gel spheres were transferred to pure dioxane for further processing. The ZrO_2_ aerogel spheres were obtained by solvent exchange into acetone, followed by supercritical CO_2_ drying. The resulting spheres closely resemble mm‐sized glass pearls (Figure 1a). After calcination to remove residual organics and increase mechanical strength, the spheres retained high optical transparency, high porosity (∼92%), and a large surface area (∼244 m^2^ g^−^^1^) (Table S3). In contrast, conventional ZrO_2_ powder supports typically exhibit surface areas between 30 and 150 m^2^ g^−^^1^ and are opaque [43, 44, 45].

ZrO_2_ is a widely used catalyst support, valued for its ability to stabilize small, highly active metal nanoparticles by suppressing sintering. It is used as a catalyst support in methanol synthesis, where it enables low‐temperature operation and facilitates the co‐feeding of CO_2_ in the syngas [46, 47]. More recently, ZrO_2_ has been identified as one of the most effective supports for direct CO_2_ hydrogenation with H_2_, enabling methanol synthesis without reliance on fossil‐derived syngas [48, 49]. State‐of‐the‐art CO_2_ hydrogenation catalysts typically pair an active metal phase (e.g., Ni, Pt, Pd), which promotes H_2_ dissociation and CO_2_ activation, with ZrO_2_ or In_2_O_3_ supports that improve stability and selectivity [49]. Building on this, we first loaded Pd on ZrO_2_ aerogel spheres and eventually introduced indium to study its influence on stability and selectivity.

Conventional oxide‐supported catalysts are typically synthesized by scalable methods such as wet impregnation, co‐precipitation [50], and flame‐spray pyrolysis [51]. Among these, wet impregnation is the most compatible with aerogels. However, standard protocols rely on solvent evaporation to deposit the metal species, which is not feasible for aerogels. Rewetting a dried gel collapses the pore network through capillary stresses. Instead, impregnation must be carried out on wet gels prior to supercritical drying [34, 35]. Earlier approaches often employed organic solvents rather than water, likely to prevent swelling or disintegration of the wet gels. This strategy, however, introduces significant limitations. Many metal salts exhibit low solubility in solvents such as ethanol or isopropanol, restricting achievable metal loadings, while others are entirely insoluble and cannot be deposited. Soluble metal‐organic precursors offer an alternative, but their high cost may limit industrial application.

To address these limitations, we employed strong electrostatic adsorption (SEA) using inexpensive, water‐soluble metal salts. SEA relies on electrostatic interactions between the charged surface of the support and oppositely charged metal‐ligand complexes, ensuring stable attachment throughout solvent exchange and supercritical drying with CO_2_. This approach is well established for powder catalysts, yielding highly dispersed active phases [52], and even enabling controlled alloying of multimetal systems [53]. Successful implementation requires tuning both the surface charge of the support and the speciation of the metal complex, typically by adjusting solution pH and selecting appropriate ligands. Ammonia is frequently used for this purpose because it is inexpensive, forms stable, positively charged complexes with many metals, and its basicity induces a negative surface charge on most oxide supports, thereby enabling strong electrostatic adsorption. In addition, ammonia is readily removed during calcination, leaving only the metal species without residual contaminants. However, ammonia is incompatible with some metal ions such as In^3+^ and Fe^3+^. For example, when ammonia is added to aqueous indium salts (e.g., indium nitrate), it immediately precipitates indium hydroxide, which cannot be redissolved by ammonia complexation. To avoid (hydr‐)oxide formation, impregnation must instead be carried out under neutral or acidic conditions. ZrO_2_ surfaces are positively charged at acidic pH values, necessitating the use of negatively charged metal complexing agents [54].

We therefore selected ethylenediaminetetraacetic acid (EDTA), which forms stable, negatively charged metal complexes while preventing hydroxide formation. Beyond this role, EDTA is an inexpensive and versatile complexing agent that can coordinate a wide range of catalytically relevant metals, offering a broadly applicable route for incorporating diverse metals [36, 55]. Importantly, it is fully removed during calcination, leaving no residues on the catalyst surface. Dissolving transition metal nitrates typically produces acidic solutions [56], and the addition of EDTA (itself an acid) further decreases the pH, limiting its solubility [57]. Efficient dissolution, therefore required deprotonation of EDTA by increasing the pH with a base. In this alkaline environment, EDTA readily complexes the metal ions, suppressing hydroxide precipitation even up to pH ∼11. To optimize electrostatic interactions, the surface charge of the ZrO_2_ nanoparticles was characterized by zeta potential measurements, which revealed an isoelectric point near pH 8 (Figure S3). The maximum positive charge was observed around pH 5, so impregnation was carried out slightly below this value to counteract the observed upward drift in pH during adsorption.

The uptake of Pd‐EDTA complexes was visually apparent. Upon immersion of the wet gels into the precursor solution, the spheres gradually acquired the yellow color of the complex, while the surrounding solution became transparent. After solvent exchange and supercritical drying, the aerogel spheres remained translucent with a faint yellow tint. Thermogravimetric analysis (TGA) of the dried Pd/ZrO_2_ (1.5 wt.% Pd) aerogel revealed a pronounced mass loss between 220 C and 300°C, consistent with literature reports for EDTA decomposition (Figure S8) [58]. Calcination at 300°C therefore removed the complexing agent and yielded darker brown spheres, reflecting a change in the coordination environment of Pd on the ZrO_2_ surface (Figure 1b). X‐ray photoelectron spectroscopy (XPS) analysis of a representative catalyst prior to calcination confirmed the presence of N and C species on the surface, consistent with residual EDTA (Table S5). After calcination, the N signal was no longer detected, while the remaining carbon content decreased to a low level attributable to adventitious carbon from air exposure. Changes in the coordination or oxidation state of Pd could not be assessed by XPS, as the Pd 3d signal overlaps with the much more intense Zr peaks (Figure S18).

The calcination of the aerogel‐based catalyst also resulted in moderate macroscopic shrinkage, with the average sphere diameter decreasing from ∼1.2 to ∼1.0 mm. Examination of crushed aerogel fragments by light microscopy revealed a color gradient from the dark brown outer surface toward a transparent core (Figure S7), indicating that the 3.5 h impregnation produces an egg‐shell type Pd distribution with a shell thickness of approximately 100 µm. Such a configuration is advantageous when the support limits mass transport and the active phase is costly [50]. Scanning transmission electron microscopy (STEM) and STEM‐energy dispersive X‐ray spectroscopy (EDX) mapping of the fragments showed no evidence of Pd clusters or nanoparticles, with the Pd signal uniformly distributed across the ZrO_2_ network (Figure S13). This indicates that calcination at 300°C and the concomitant removal of the EDTA do not induce significant Pd sintering on the ZrO_2_ surface.

To better understand the deposition behavior, we systematically varied the Pd‐complex concentration in the impregnation solution, defining complete deposition of the available Pd as the “nominal Pd loading” (1–6 wt.%). X‐ray fluorescence (XRF) analysis of the calcined aerogels showed that complete deposition occurred only at low nominal loadings (∼1 wt.%), likely because the small amount of Pd rapidly adsorbed on the outer surface of the aerogel spheres. At higher nominal loadings, only partial incorporation of Pd was achieved, indicating that slow diffusion of the Pd‐EDTA complexes into the interior of the gel limited overall uptake (Figure 1d). Since the impregnation time was held constant, there was not enough time for all Pd species to reach available adsorption sites, leaving a fraction of Pd in solution. As a result, a Pd loading gradient formed within the spheres, with higher loadings near the surface and lower loadings toward the core. This observation is consistent with the optical microscopy results described above (Figure S7). The most concentrated Pd^2+^ impregnation solution yielded a Pd content of 2.5 wt.%. If desired, higher loadings could likely be achieved by extending the impregnation time or by increasing the precursor concentration to enhance the diffusional driving force into the gel.

Since metallic Pd is required both as the active phase and as an efficient photothermal nanoheater for CO_2_ reduction, the reducibility of Pd species was evaluated by H_2_ temperature‐programmed reduction using a mixture of H_2_ and N_2_ (H_2_‐TPR, Figure 1e). Bare ZrO_2_ was tested as a control and showed no reduction below 500°C. In contrast, Pd/ZrO_2_ exhibited reduction onset near 120°C, continuing up to ∼450°C. For comparison, Pd deposited on SiO_2_ powders via SEA was reduced fully between 120 C–250°C [53]. The broader reduction profile observed for Pd/ZrO_2_ indicates stronger metal‐support interactions, which make part of the Pd phase more difficult to reduce. STEM‐EDX mapping of reduced Pd/ZrO_2_ (200°C, H_2_/N_2_ = 10/10 SCCM, 1 h) revealed well‐dispersed Pd nanoparticles with sizes ranging from 0.5 to 3 nm and an average diameter of ∼1.5 nm (Figure 1f and Figure S15). Such small particles are significantly below the sizes commonly obtained via precipitation‐deposition (∼4 nm) [59], dry impregnation (∼4 nm) [52], or conventional wet impregnation (∼3 nm) [60], and are comparable to the smallest values reported for SEA‐based syntheses (∼1.5 nm) [52, 53]. This indicates that our protocol yields a catalyst whose Pd particle size and surface area are competitive with the best reported for highly established thermocatalyst systems.

The catalytic activity of the Pd/ZrO_2_ aerogel catalyst was first evaluated under purely thermocatalytic conditions in the absence of illumination. The catalyst was reduced at 300°C for 1 h, after which the reaction feed was introduced, and product formation was monitored at temperatures between 300 C and 180°C, holding each temperature for 3 h. Subsequently, an Arrhenius plot was constructed, yielding an apparent activation energy of 71.8 kJ mol^−1^ (Figure S9). This value is consistent with literature reports for Pd‐based reverse water‐gas shift (RWGS) catalysts [61], confirming that the Pd/ZrO_2_ aerogel exhibits appreciable thermal activity even within the low‐temperature regime of this reaction.

**FIGURE 2:**
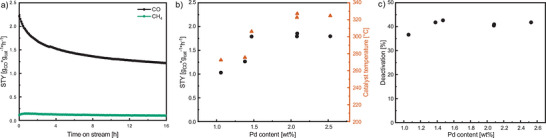
Photothermal catalytic performance of Pd/ZrO_2_ aerogel catalysts. (a) STY of CO and CH_4_ over time on stream, showing rapid initial deactivation followed by a marked decline in deactivation rate. (b) Initial CO space‐time yield (STY, left axis, black dots) and corresponding average catalyst bed temperature measured by IR thermography (right axis, orange triangles) as a function of Pd loading. (c) Catalyst deactivation, expressed as the percentage decrease in CO STY after 16 h on stream relative to the initial rate, for different Pd contents. Reaction conditions: light intensity 4.8 W cm^−2^; total pressure 0.1 MPa; feed composition H_2_/CO_2_/N_2_ = 9:9:1 SCCM; gas hourly space velocity (GHSV) ∼3700 h^−1^; CO_2_ conversion ∼5%.

Photothermal performance was evaluated after reducing the catalyst at 200°C under H_2_, forming Pd nanoparticles that act as strong broadband light absorbers and give the aerogel spheres their dark black appearance (Figure 1c). After cooling the reactor to ∼40°C and introducing the reaction feed, the high‐power LED was gradually ramped to its maximum output (4.8 W cm^−2^). All samples exhibited a high initial CO space‐time yield (STY), likely reflecting the excellent initial dispersion of Pd nanoparticles and the correspondingly large active surface area. The activity then declined rapidly over the first ∼4 h on stream, after which the rate of deactivation slowed markedly and the activity decreased much more gradually with time (Figure 2a). To examine the effect of Pd loading, the initial STY was plotted as a function of Pd content and compared with the corresponding average catalyst temperatures measured by infrared (IR) thermography (Figure 2b). The STY increased with Pd loading up to ∼1.5 wt.% Pd, above which it appeared to saturate. The temperature trend closely mirrored the activity: higher average catalyst temperatures corresponded to higher CO production rates. For the graph, the average temperature of all granules was used, though the IR thermographs revealed slight temperature variations between granules due to differences in heat retention within the packed bed (Figure S22).

In the photothermal Pd/ZrO_2_ catalyst, the Pd nanoparticles act as nanoheaters embedded within the transparent ZrO_2_ network. At low Pd loadings, the number of nanoheaters is insufficient to capture all incident light, resulting in lower catalyst temperatures and reduced activity. Once the nanoheater density is sufficient to absorb nearly all incoming light, further increases in Pd content do not raise the average catalyst temperature. Increasing the nanoheater density beyond this point may limit light penetration to the outer layers of the spheres. Although the light energy is then concentrated in a thinner volume, no additional temperature increase is observed, which could be due to enhanced heat losses to the surroundings. Another possible explanation is that higher Pd loadings reduce particle dispersion and increase average Pd particle size, therefore lowering nanoheater density. Because Pd dispersion was not directly quantified as a function of loading, this explanation remains tentative. Nevertheless, these findings highlight a key design principle: in photothermal catalysts where the active metal also serves as the light absorber, an optimal metal loading exists, beyond which further increases do not improve activity.

An alternative strategy to enhance activity is to reduce catalyst deactivation. Here, deactivation was quantified as the difference between the initial CO STY and the STY after 16 h on stream, normalized to the initial value. The resulting deactivation as a function of Pd loading is shown in Figure 2c. The degree of deactivation was remarkably consistent (∼42%) across most Pd loadings, with only the lowest‐loading sample (1.1 wt.%) showing slightly lower deactivation (∼37%). STEM‐EDX analysis of Pd/ZrO_2_ after 18 h on stream revealed a pronounced increase in particle size compared to the freshly reduced catalyst. The average Pd particle size increased from ∼1.5 ± 0.5 to ∼3 ± 1.7 nm, confirming substantial sintering during reaction (Figures S15–S17). Such growth reduces the available active surface area, providing an explanation for the observed deactivation. The lower deactivation observed in the lowest‐loading sample is consistent with the known tendency for smaller amounts of active metals to sinter less. This interpretation is consistent with previous reports on Pd/ZrO_2_ [44] and other CO_2_‐conversion catalysts [62, 63, 64], where active phase sintering has been cited as a dominant deactivation mechanism.

Another frequently reported deactivation pathway in RWGS catalysts is the deposition of carbonaceous species on the active surface, commonly referred to as coking [65, 66, 67]. In related systems, such as methane dry reforming catalysts, XPS has been used to identify coking through an increase in surface carbon content [68]. In contrast, XPS analysis of a representative catalyst from this study before and after reaction did not show an increase in carbon content (Table S5), indicating that coking does not contribute significantly to catalyst deactivation under the applied conditions. In addition to active metal sintering, structural changes of the aerogel support may also contribute to catalyst deactivation. Light microscopy revealed that the average sphere diameter decreased from ∼1.0 to ∼0.9 mm after 18 h of photothermal catalysis (Figure S6), corresponding to a ∼19% reduction in cross‐sectional area. Because of the spherical geometry, this shrinkage does not translate directly into a 19% reduction in absorbed photon energy, but the decrease in light harvesting is nevertheless significant. STEM and Brunauer–Emmett–Teller (BET) surface area  analyses indicate that the shrinkage arises from densification and sintering of the ZrO_2_ backbone, with BET showing a 23% surface area loss (Table S3) and STEM revealing ZrO_2_ nanoparticle growth from ∼3.5 to ∼5.8 nm (Figures S10 and S11). Powder X‐ray diffraction (XRD) could not reliably quantify crystallite size due to the coexistence of tetragonal and monoclinic ZrO_2_ in this size regime, complicating Scherrer analysis (Figure S12). However, the intensity of monoclinic peaks increased during catalysis, consistent with growth‐induced phase evolution [69]. Supporting densification and grain growth increases the thermal conductivity of the aerogel, thereby enhancing heat loss to the surroundings. This relationship is further supported by comparative experiments on aerogel samples with different degrees of densification, as discussed in a later section. Together with the reduced light absorption due to the shrinkage of the spheres, these two effects led to an appreciable ∼11°C decrease in the average granule temperature after extended time on stream. While support densification is also observed in conventional thermal catalysis, the resulting drop in catalyst temperature under illumination represents a deactivation pathway unique to photothermal operation.

**FIGURE 3:**
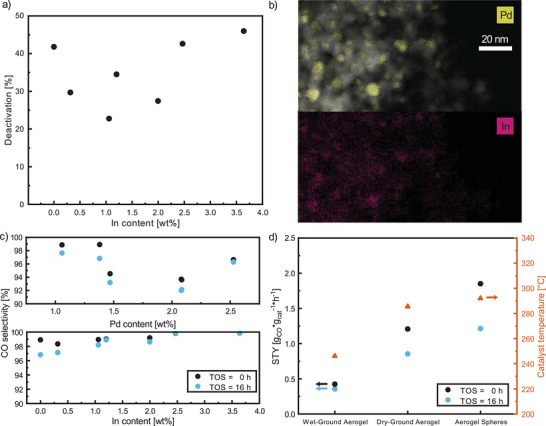
Characterization and performance of aerogel‐supported Pd/ZrO_2_ and PdIn/ZrO_2_ catalysts. (a) Catalyst deactivation, expressed as the percentage decrease in CO STY after 16 h on stream relative to the initial rate, for PdIn/ZrO_2_ catalysts with different In contents and constant Pd content (∼1.4 wt.%). (b) High‐angle annular dark‐field (HAADF)‐STEM images and STEM‐EDX maps of spent PdIn/ZrO_2_ (1.4 wt.% Pd, 1.1 wt.% In, 18 h on stream) showing the aerogel structure decorated with Pd nanoparticles (∼3.5 nm, top) and partial co‐location of In with Pd nanoparticles (bottom). (c) CO selectivity as a function of Pd content for Pd/ZrO_2_ (top) and as a function of In content for PdIn/ZrO_2_ at constant Pd content (1.4 wt.%, bottom). CO selectivity for the 2.5 and 3.6 wt.% In samples did not change after 16 h, resulting in overlapping scatter points. (d) Initial and 16 h time‐on‐stream (TOS) CO space‐time yield (STY, left axis, spherical dots) and corresponding initial average catalyst bed temperature (right axis, triangles) of PdIn/ZrO_2_ (1.3 wt.% Pd, 1.2 wt.% In) catalysts in different forms: intact aerogel spheres, dry‐ground aerogel (fragmented aerogel), and wet‐ground aerogel (collapsed aerogel resembling a conventional powder catalyst). Reaction conditions: light intensity 4.8 W cm^−2^; total pressure 0.1 MPa; feed composition H_2_/CO_2_/N_2_ = 9:9:1 SCCM; gas hourly space velocity (GHSV) ∼3700 h^−1^; CO_2_ conversion ∼5%.

Strategies to improve the stability of CO_2_ conversion catalysts often focus on mitigating active‐phase sintering, for example, by co‐depositing secondary metals that inhibit particle growth [70]. Araújo et al. reported that co‐depositing indium on Pd/ZrO_2_ for CO_2_ hydrogenation to methanol effectively impeded Pd sintering [60]. Our wet‐impregnation approach was designed to enable the deposition of such secondary metals, including indium. A constant Pd loading (1.4 wt.%) was first adsorbed via strong electrostatic adsorption, followed by the deposition of varying amounts of In‐EDTA. XRF analysis (Table S4) confirmed successful deposition of both metals on the ZrO_2_ aerogels. The effect of indium content on photothermal performance was then assessed under the same reaction conditions as the monometallic Pd/ZrO_2_ aerogel. Small additions of indium (0.3 wt.%) led to a modest ∼20% increase in the initial CO space‐time yield (STY), which correlated with a ∼13°C rise in average catalyst temperature, likely arising from the higher total metal content and the associated enhancement in light absorption (Figure S23). Further increases in indium content did not lead to additional temperature or activity gains, and the STY plateaued at this level across the range of tested In loadings. Interestingly, indium addition had a notable effect on the catalytic stability. Deactivation was progressively reduced with increasing indium content, reaching a minimum of 25%–30% at In loadings of 1–2 wt.% (Figure 3a). At higher indium loadings, the beneficial effect of In on catalyst stability diminished, and the degree of deactivation approached that of the monometallic Pd catalyst. STEM‐EDX mapping of spent catalysts indicated partial co‐location of Pd and In (Figure 3b), consistent with previous reports on PdIn/ZrO_2_ systems [60, 71, 72]. This conclusion is further supported by the H_2_‐TPR profile, which deviates from a simple superposition of the monometallic samples and instead displays distinct reduction features (Figure 1e). Particle size analysis based on STEM‐EDX images showed that the average Pd particle size was comparable to that of the monometallic catalyst (3.5 ± 1.3 vs. 3.0 ± 1.7 nm, Figure S25), and the relative BET surface area loss was likewise similar (21% vs. 23%, Table S4). The slightly greater reduction in surface area observed for the bimetallic samples likely results from their higher total metal loading (2.41 wt.% vs. 1.47 wt.%) [73]. Since STEM‐based particle size analysis indicates that sintering is not significantly suppressed in the PdIn catalysts, the improved stability is more likely related to alternative mechanisms, such as electronic modification of Pd sites or ensemble effects [44, 60, 74]. Further studies will be required to identify the dominant mechanism.

For long‐term stability measurements, an indium content of 1.2 wt.% was selected as it provides a balance between catalytic stability and CO selectivity. As shown in the preceding experiments, higher indium loadings resulted in increased deactivation, while lower indium contents led to a decrease in CO selectivity to below 98%. The CO space–time yield (STY) vs. time on stream is shown in Figure S24. The catalyst exhibited a pronounced initial deactivation over the first 7 h on stream, during which the activity decreased to 55% of its initial value (corresponding to an average deactivation rate of 6.4% per hour). Following this initial period, the deactivation rate decreased substantially and continued to decline with increasing time on stream. For example, between 60 and 100 h on stream, the activity decreased by only 4.5% relative to the initial value (0.1% per hour). While a fully stable plateau was not reached within the investigated timeframe, the deactivation rate after 110 h remained very low, indicating that no additional or fundamentally different deactivation processes became apparent at longer time scales. Previous studies on PdIn/ZrO_2_ for thermal catalysis have reported that indium addition enhances both the selectivity and stability for methanol synthesis via CO_2_ hydrogenation [49, 60, 75, 76]. Because methanol synthesis generally requires high pressures [77], which were not accessible with our reactor system, methanol formation was not observed. Under ambient‐pressure CO_2_ hydrogenation conditions, CO is typically the desired product because it can be directly utilized in downstream processes such as Fischer‐Tropsch synthesis. Methane, in contrast, forms through parasitic hydrogenation pathways and represents a terminal product that cannot be further valorized and is thus undesired. While other products have been reported in CO_2_ reduction catalysis, in our experiments only CO and CH_4_ were detected, as confirmed by gas chromatographic analysis (Figures S20 and S21). The CO selectivity of monometallic Pd/ZrO_2_ aerogels is shown in the upper panel of Figure 3c, comparing fresh catalysts and samples after 16 h on stream. CO selectivity was high for lower Pd‐loading samples (∼98%), while higher‐loading samples showed slightly lower selectivity (∼93%). Across all compositions, CO selectivity declined modestly (∼1.5%) over time, because the CO STY decreased faster compared to the CH_4_ STY. By contrast, the co‐deposition of In with Pd had a pronounced effect on selectivity (Figure 3c, bottom). CO selectivity increased monotonically with In content, reaching >99% for In loadings above 1.2 wt.%. The improved CO selectivity upon In addition likely arises from electronic and geometric effects associated with Pd‐In co‐location. Electron donation from In reduces the Lewis acidity of nearby Pd atoms, weakening CO adsorption. The presence of In next to Pd also disrupts contiguous Pd ensembles, decreasing the number of strongly binding multicoordination sites and increasing the fraction of weakly bound linear sites. Both effects favor CO desorption over full hydrogenation to methane, consistent with previous mechanistic studies of Pd‐In catalysts [44, 72, 78].

A central aim of this work was to demonstrate that transparent aerogel supports are particularly well suited for photothermal catalysis, where efficient light absorption and retention of the generated heat are critical. Minimizing optical losses due to reflection and scattering ensures that a larger fraction of the incident light is converted into heat within the catalyst. To probe these optical effects, PdIn/ZrO_2_ aerogels were gently ground in a mortar to produce a powder composed of smaller aerogel fragments, referred to here as the “dry‐ground aerogel.” The resulting dry‐ground aerogel exhibited a visually apparent increase in reflectance, which was confirmed by total reflectance measurements (Figures S26–S27). Infrared (IR) thermography during photothermal catalysis (4.8 W cm^−2^) indicated that the surface temperature of the dry‐ground aerogel was approximately 10°C lower than that of the aerogel spheres, likely due to reduced light absorption associated with the ∼11% higher reflectance. Based on thermocatalytic (dark) experiments with intact PdIn/ZrO_2_ aerogel spheres (Figure S19), this temperature difference would be expected to cause a ∼25% decrease in CO STY. In reality, the dry‐ground aerogel exhibited an even larger drop of about 35% (Figure 3d). This stronger‐than‐expected decrease likely reflects the fact that thermography predominantly probes the surface, whereas the bulk of the packed powder bed may have remained significantly cooler. Increased light scattering in the fragmented aerogel particles likely restricted photothermal heating to the upper portion of the bed, reducing the effectively heated volume and thereby lowering the overall CO STY. In terms of stability, the dry‐ground aerogels deactivated by ∼29%, compared to ∼34% for the intact spheres, consistent with the lower photothermal temperature mitigating sintering and densification effects.

Beyond optical effects, the thermal transport properties of the support are equally important, as they govern how efficiently the generated heat is retained within the catalyst bed. To further probe the role of thermal conductivity, another portion of the PdIn/ZrO_2_ aerogel spheres was thoroughly ground in ethanol, collapsing the aerogel network via capillary forces while maintaining the catalyst composition. This sample is referred to here as the “wet‐ground aerogel”. Wet‐grinding produced a reference material whose structure more closely resembled a conventional powder than an aerogel. The wet‐ground aerogel showed an increase in reflectance, with total reflectance slightly higher than that of the dry‐ground sample (Figures S26). N_2_ physisorption confirmed the loss of the aerogel's characteristic mesoporosity and revealed a ∼10% decrease in BET surface area likely due to tighter packing of the ZrO_2_ nanoparticles (Figure S28 and Table S6). This denser microstructure increases the effective thermal conductivity of the material, leading to enhanced heat dissipation and lower maximum temperatures under photothermal illumination. Consistently, the wet‐ground aerogel reached a temperature approximately 44°C lower than the intact spheres during photothermal operation (Figure 3d). This difference cannot be explained by the modest increase in reflectance alone and instead indicates increased conductive heat losses associated with the denser structure. This interpretation is further supported by cooling experiments (Figure S29), in which aerogel spheres and ground reference samples were heated to 300°C and their temperature decay was monitored. The ground samples cooled significantly faster than the aerogel spheres, indicating a higher effective thermal conductivity, in agreement with their lower steady‐state temperatures under illumination. The resulting lower operating temperature caused an approximately fourfold decrease in CO production. Although the wet‐ground aerogel exhibited the lowest deactivation over 16 h on stream (16%), its overall activity remained far below that of the aerogel spheres, achieving only ∼29% compared to the aerogel spheres. CO selectivity remained above 99% for all samples, indicating that the observed differences in activity arise primarily from changes in the heated volume and achievable catalyst temperature rather than differences in intrinsic selectivity.

Comparison with powdered reference materials, whether partially fragmented (dry‐ground aerogel) or fully collapsed into a nanopowder (wet‐ground aerogel), clearly demonstrates that the intact aerogel spheres provide a structurally superior platform for photothermal catalysis. Their unique combination of high optical transmittance and exceptionally low thermal conductivity enables more efficient light absorption and heat retention than achievable with conventional powder‐like structures. While further optimization of the PdIn/ZrO_2_ composition remains possible, particularly with respect to sintering resistance, it already showed strong performance. After 110 h on stream, it maintained a stable STY of approximately 0.7 g_CO_ g_cat_^−1^ h^−1^ (25 mmol_CO_ gcat^−1^ h^−1^) placing it at the upper end of reported photothermal RWGS catalysts, which typically deliver around 0.1–0.6 g_CO_ gcat^−1^ h^−1^ under broadly similar reaction conditions [79, 80, 81, 82, 83].

## 3 Conclusion

We developed a wet‐impregnation strategy to produce transparent ZrO_2_ aerogel spheres loaded with catalytically active metals, enabling efficient photothermal CO_2_ reduction. Negatively charged Pd‐ and In‐EDTA complexes were deposited onto the oppositely charged ZrO_2_ surface via strong electrostatic adsorption. Calcination at 300°C removed the complexing agent without inducing metal sintering, and the metals were subsequently reduced to form well‐dispersed Pd and PnIn nanoparticles. These nanoparticles functioned both as the active phase and as photothermal nanoheaters, while the transparent ZrO_2_ backbone enabled deep light penetration and low thermal conductivity. Under identical illumination, the aerogel‐supported catalysts reached temperatures up to 300°C, over 40°C higher than the powder catalysts tested under the same conditions, resulting in nearly fourfold higher activity.

Varying the Pd content revealed a regime in which further increases in loading no longer improved activity, indicating that once all incident light is absorbed, additional nanoheaters do not enhance activity. The catalysts exhibited an initial rapid deactivation during the first few hours on stream, after which their performance stabilized. This initial deactivation likely originated from sintering of the Pd phase under reaction conditions, a phenomenon also commonly observed in thermally driven RWGS catalysts [84]. Microscopy and BET analysis showed densification of the ZrO_2_ support during reaction, likely increasing thermal conductivity and reducing the achievable temperature under illumination. Co‐deposition of In significantly mitigated deactivation and improved CO selectivity to above 99%.

The wet‐impregnation approach demonstrated here provides a versatile route to incorporate metals into aerogels toward more active, selective, and stable catalysts. This work shows that aerogels are highly promising supports for photothermal catalysis. Their transparency and low thermal conductivity enable higher catalyst temperatures and improved utilization of the catalyst bulk. Combined with the versatile metal loading strategy presented here, these materials open new opportunities to design photothermal catalysts capable of reducing the carbon intensity of chemical production and supporting the transition toward a net‐zero chemical industry.

## 4 Methods

### 4.1 Chemicals and Materials

Ethanol absolute for analysis (>99.8%), benzyl alcohol puriss. p.a. (≥99.0%), 1,4‐dioxane puriss. p.a. (≥99.5%), nitric acid, puriss. p.a. (≥65%), ethylenediaminetetraacetic acid (EDTA ≥99%), indium(III) nitrate hydrate (99.9% trace metals basis), and acetone (>99.8%) were purchased from Merck/Sigma Aldrich. Hydrochloric acid, fuming (>37% AnalR NORMAPUR), and ammonia 25% analytical reagent (AnalR NORMAPUR) were received from VWR International AG. Zirconium(IV) chloride (99.5+% metals basis) was purchased from Thermo Scientific—Alfa Aesar (ALF), ethyl acetate from Thommen‐Furler AG, heptanes (mixture of isomers) from Fischer Scientific, silicone oil (Bluesil Fluid 47 V 350) from Silitech AG, and palladium(II) nitrate hydrate (99.9% Pd) from abcr GmbH. Carbon dioxide 4.5, nitrogen 5.0, and hydrogen 5.0 were purchased from PanGas AG Switzerland. All chemicals were used as received without further purification.

### 4.2 Synthesis of ZrO_2_ Nanoparticles

Zirconium dioxide (ZrO_2_) nanoparticles were synthesized following a recently reported non‐aqueous procedure [41]. Zirconium(IV) chloride (ZrCl_4_, 10.9 g, 0.047 mol) was placed in a vial cooled in an ice bath. Pre‐cooled ethanol (10.9 mL, 0.187 mol, −20°C) was added dropwise to the ZrCl_4_ under stirring to control the exothermic reaction. Separately, benzyl alcohol (260 mL) was preheated to 180°C in a 500 mL round‐bottom flask using a heating block. The ethanolic ZrCl_4_ solution was added dropwise to the hot benzyl alcohol under vigorous stirring (450 rpm), causing the temperature to decrease to 160°C. The reaction mixture was then maintained at 180°C for 30 min, yielding a white precipitate. Fuming hydrochloric acid (14.65 mL) was then carefully added, and after 1 min the reaction was quenched by immersion of the flask in an ice bath.

To wash and collect the nanoparticles, they were precipitated by adding 30 mL of a 1:1 (v/v) ethyl acetate/heptane solution to 20 mL of the reaction mixture. The precipitated particles were recovered by centrifugation (4000 rpm, 20 min) and subjected to two additional washes with ethyl acetate (2 × 45 mL) and heptanes (3 × 45 mL), with centrifugation (4000 rpm, 5 min) after each wash to collect the solids.

To obtain an aqueous dispersion, the precipitate was initially suspended in heptanes (20 mL), followed by the careful addition of water (8 mL, Mili‐Q) without shaking or stirring. The two‐phase system was then centrifuged for 90 s at 4000 rpm to transfer the nanoparticles into the aqueous phase. The aqueous fraction was carefully removed with a syringe and combined with other aliquots to enable subsequent processing at a larger scale. This combined dispersion was placed in an open glass bottle for at least 24 h to allow residual heptanes to evaporate and for any agglomerated particles to re‐disperse, yielding a clearer dispersion. Prior to dialysis, the dispersion was filtered using a syringe filter (PALL Acrodisc 32 mm with 0.2 µm Supor membrane). Dialysis was performed against Mili‐Q water for 9 h using a Spectra/Por Float‐A‐Lyzer with a molecular weight cut‐off (MWCO) of 3.5‐5 kD. After dialysis, the dispersion was concentrated at 30°C and 10 mbar using a rotary evaporator to achieve a final nanoparticle concentration of 160 mg/mL.

### 4.3 Fabrication of ZrO_2_ Gels Spheres

Gelation was initiated by adding 0.75 mL of dioxane to 0.5 mL of ZrO_2_ nanoparticle dispersion under vortexing. The resulting pre‐gel was transferred to a 5 mL syringe, air bubbles were removed, and after 10 min at room temperature the syringe was cooled in an ice bath to slow gelation. Meanwhile, a 50 mL vial containing a silicone oil phase above a 4:1 (v/v) dioxane/water mixture was heated to 65°C. The pre‐gel was extruded dropwise into this two‐phase system using a syringe pump, with the syringe maintained at 0°C. Upon contact with the silicone‐oil phase, droplets rapidly formed spherical beads that fully solidified in the underlying dioxane‐rich phase. The silicone oil was then removed, and the gel spheres were transferred to dioxane. To be able to store them for later use, the spheres were gradually transferred from dioxane to aqueous ammonia (pH 10.6). Careful stepwise solvent exchange was used to minimize shrinkage of the gel spheres. The ZrO_2_ gels could then be stored in ammonia solution for up to several weeks prior to further processing.

### 4.4 Impregnation of ZrO_2_ Gel Spheres

A palladium (Pd) stock solution was prepared by dissolving both palladium nitrate and ethylenediaminetetraacetic acid (EDTA) at a 1:1 molar ratio in an ammonia solution (pH ∼ 10.7). After several hours of stirring the pH was first adjusted back to ∼10.7 with ammonia to promote full EDTA dissolution, then lowered to 3.5 with nitric acid, yielding a stock solution with a Pd concentration of 0.2 mg/mL (1.88 µmol/mL). An identical procedure was used to prepare the indium‐EDTA solution from the corresponding nitrate precursor. Aliquots of these stock solutions were subsequently diluted with nitric acid (pH 3.8) to obtain impregnation solutions of varying concentrations. For the detailed preparation of the impregnation solutions please refer to the Supporting Information. Prior to impregnation, the ZrO_2_ gel spheres were transferred from the storage solution to neutral water (45 mL), followed by exchange into nitric acid (pH 3.0, 45 mL), and finally back to neutral water (45 mL), yielding a suspension with a final pH of 3.8. At this slightly acidic pH, the ZrO_2_ nanoparticles in the gel carry a positive surface charge, which facilitates the adsorption of negatively charged EDTA‐metal complexes.

For impregnation, the aqueous phase was replaced with 45 mL of a yellow Pd‐EDTA solution. The sealed vial was placed on a roller to ensure homogeneous mixing. After 2 h, the pH of the solution had increased and was readjusted to 3.5 with 100 µL of nitric acid (pH 1.45). After an additional 1.5 h on the roller, the solution became colorless while the ZrO_2_ spheres acquired the characteristic yellow of the Pd complex. For indium loading, a consecutive impregnation step was performed using an In‐EDTA solution.

After impregnation, the spheres were transferred to acetone and washed twice with acetone prior to supercritical drying. In the supercritical dryer (E3100, Quorum Technologies), acetone was gradually replaced with liquid CO_2_, followed by supercritical drying at 42°C and 100 bar. Finally, the ZrO_2_ aerogel spheres were calcined at 300°C in air for 24 h (1°C min^−1^ ramp) to remove residual organics and EDTA.

Reference materials were prepared by gently grinding 40 mg of calcined aerogel catalyst in a mortar, either dry or wet (ethanol, 5 mL) to produce different catalyst morphologies with identical composition.

### 4.5 Characterization

Scanning transmission electron microscopy (STEM) was performed on a FEI Talos F200X microscope operated at 200 kV and equipped with a Super‐X EDXS detector for elemental analysis. Aerogel samples were fragmented with a scalpel, dispersed in ethanol (1–10 mg mL^−^^1^), and drop‐cast onto S166 lacey carbon grids. Temperature‐programmed reduction (TPR) measurements were carried out using a MicrotracBEL BelCat II catalyst analyzer equipped with a thermal conductivity detector (TCD). Approximately 50 mg of sample was loaded into the quartz sample cell, pretreated under flowing Ar (50 mL min^−1^) at 300°C for 60 min, and then cooled to 40°C. The TPR experiment was performed under a flow of 10 vol % H_2_ in Ar (30 mL min^−1^) while ramping the temperature to 500°C at 5°C min^−1^. X‐ray fluorescence (XRF) was used to determine the metal content of ground aerogel samples, using a Rigaku ZSX Primus IV spectrometer equipped with a 4 kW Rh source and LiF(200), Ge, PET, and RX26 crystal detectors.

Photocatalytic testing was performed using a custom‐built reactor system, comprising a gas flow controller (Alicat Scientific), a heatable photoreactor (Figure S1), a white LED illumination system (77 W) (Figure S2), and a micro‐gas chromatograph (Micro GC Fusion, Inficon) equipped with three modules (Rt‐Molsieve 5A with Rt‐Q‐Bond Backflush, Rt‐Q‐Bond, and Rt‐U‐Bond) and TCD detectors. For testing, ∼19 mg (∼0.32 cm^3^) of aerogel granules were loaded into a 10 mm diameter aluminum cup. The cup facilitated insertion and removal of the granules from the 15 mm diameter, 3.5 cm^3^ reactor chamber and was thermally insulated from the chamber using a 1 mm thick glass fiber mat. The chamber could be sealed with a CaF_2_ window, enabling mid‐infrared transmission for catalyst bed temperature monitoring using an IR camera (IRCAM Millenium 327k S/M). Catalyst activation was carried out under a 1:1 mixture of N_2_ and H_2_ at 20 cm^3^ STP min^−1^ (SCCM) while heating to 200°C. The reducing atmosphere was maintained for 1 h, after which the system was cooled to ∼60°C and switched to the reaction mixture (CO_2_:H_2_:N_2_ = 9:9:2, total flow 20 SCCM), corresponding to a gas hourly space velocity of ∼3700 h^−^^1^ (∼63 L g^−^^1^ h^−^^1^). Thermal activation energies were determined by varying the sample temperature between 180°C and 300°C in 10°C increments, allowing 3 h of equilibration at each setpoint in the dark. Photothermal activity was assessed under concentrated white LED illumination with an intensity of 4.8 W/cm^2^ (48 suns). Additional details of the photothermal reactor setup are provided in the Supporting Information.

## Author Contributions

D.K. conceptualized the study, conducted characterization, and processed the data. L.R. synthesized all samples and aided in characterization. E.T. acquired the TEM and STEM/EDX images. D.K. and L.R. drafted the manuscript, while M.N. reviewed and edited the final version.

## Conflicts of Interest

The authors declare no conflicts of interest.

## Supporting information




**Supporting File**: advs76221‐sup‐0001‐SuppMat.docx.

## Data Availability

Data available from the corresponding author on reasonable request.
