# Supplementary material for: ZrO2 Aerogel‐Supported Pd Nanoparticles for Photothermal CO2 Reduction
Source: Adv Sci (Weinh). 2026 Jun 25:e76221. Online ahead of print. doi: 10.1002/advs.76221 (PMC13336964; doi:10.1002/advs.76221)
Supplement: Supplementary file 1 — Supporting File: advs76221‐sup‐0001‐SuppMat.docx. [file ADVS-9999-e76221-s001.docx]

Supplementary Information:

ZrO_2_ Aerogel-supported Pd Nanoparticles for Photothermal CO_2_ reduction

*David Kiwic^1^, Linard Räz^1^, Elena Tervoort^1^, Markus Niederberger^1^*

^1^Laboratory for Multifunctional Materials, Department of Materials, ETH Zurich, Vladimir-Prelog-Weg 5, 8093 Zurich, Switzerland

Table of contents

Methods 1

Detailed preparation of Pd- and In-EDTA stock solutions 1

Grinding of PdIn/ZrO_2_ aerogels to produce reference samples 3

Characterization 3

Heatable photoreactor and illumination system 6

Results and Discussion 9

Surface charge and morphology of ZrO_2_ nanoparticles 9

Light microscopy analysis of Pd/ZrO_2_ aerogel sphere size 11

Macroscopic Pd distribution throughout the ZrO_2_ aerogel spheres 13

Thermal, compositional, and structural characterization of Pd/ZrO_2_ aerogels 13

Apparent activation energy of Pd/ZrO_2_ during thermal catalysis 15

Structural analysis of ZrO_2_ aerogel backbone in Pd/ZrO_2_ catalysts 16

Pd dispersion on calcined Pd/ZrO_2_ sample 19

Pd dispersion on reduced Pd/ZrO_2_ sample 20

Pd dispersion on the spent photothermal Pd/ZrO_2_ catalyst 21

Compositional and structural characterization of PdIn/ZrO_2_ aerogel catalysts 23

Apparent activation energy of PdIn/ZrO_2_ during thermal catalysis 26

Gas chromatograms during photothermal catalysis with PdIn/ZrO_2_ 27

Infrared thermography of PdIn/ZrO_2_ aerogel spheres during photothermal catalysis 29

Photothermal activity of PdIn/ZrO_2_ catalysts 30

Long-term stability of the PdIn/ZrO_2_ catalyst in photothermal catalysis 31

Pd and In dispersion on the spent PdIn/ZrO_2_ catalyst 33

Optical, morphological and thermal properties of PdIn/ZrO_2_ aerogels compared to reference powders 35

References 40

## Methods

### Detailed preparation of Pd- and In-EDTA stock solutions

A Pd–EDTA stock solution was prepared for Pd impregnation of ZrO_2_ gel spheres. Palladium(II) nitrate hydrate (Pd(NO_3_)_2_)·xH_2_O, 101.4 mg, 0.381 mmol, certificate of analysis (CoA): x = 1.98) and ethylenediaminetetraacetic acid (EDTA) (115.4 mg, 0.395 mmol) were dissolved in 200 mL of aqueous ammonia (pH 10.63) and stirred for several hours. A glass magnetic stir bar was used, as PTFE stir bars led to the formation of an undefined black precipitate. The pH was adjusted to 10.75 with 25% aqueous ammonia to ensure complete EDTA dissolution. After dissolution, the pH was lowered to 3.8 by dropwise addition of nitric acid, yielding a final solution volume of 208 mL with a palladium concentration of 0.195 mg mL^-1^. The stock solution was stored at room temperature in a sealed glass bottle under continuous stirring until use. For catalyst preparation, aliquots of the Pd–EDTA stock solution were diluted with nitric acid solution (pH 3.8) to obtain solutions with the desired Pd concentrations (**Table S1**). ZrO_2_ gel spheres were impregnated with 45 mL of each solution according to the procedure described in the main Methods section.

**Table S1** **|** Palladium impregnation solutions and resulting Pd loadings in Pd/ZrO_2_ aerogel, determined by X-ray fluorescence (XRF).

| **Pd concentration of impregnation solution [mg mL^-1^]** | **Pd loading [wt%]** |
| --- | --- |
| 0.016 | 0.9 |
| 0.031 | 1.4 |
| 0.047 | 1.5 |
| 0.062 | 2.1 |
| 0.078 | 2.1 |
| 0.094 | 2.5 |

An In–EDTA stock solution was prepared for the sequential deposition of indium after Pd impregnation. Indium(III) nitrate hydrate (In(NO_3_)_3_·xH_2_O, 86.6 mg, 0.249 mmol, CoA: x = 2.61) and EDTA (115.4 mg, 0.250 mmol) were dissolved in 200 mL of aqueous ammonia (pH 10.63) and stirred for several hours using a PTFE magnetic stir bar. The pH was adjusted to 10.75 with 25% aqueous ammonia to ensure complete EDTA dissolution, followed by adjustment to pH 3.8 with nitric acid. The final solution volume was 207 mL, corresponding to an indium concentration of 0.1381 mg mL^-1^. The solution was stored under constant stirring in a sealed glass bottle until further use.

To prepare PdIn/ZrO_2_ aerogels, a fresh Pd–EDTA solution was prepared as described above, with a slightly higher Pd concentration (0.2215 mg mL^-1^). ZrO_2_ gel spheres were impregnated with 45 mL of a 0.018 mg mL^-1^ Pd solution, following the same procedure as for monometallic Pd samples. Immediately after Pd impregnation, a second impregnation step was carried out using In–EDTA solutions of varying concentrations to achieve different In loadings (**Table S2**).

**Table S2** **|** Indium impregnation solutions and resulting Pd and In loadings in PdIn/ZrO_2_ aerogel, determined by XRF.

| **Indium concentration of impregnation solution [mg mL^-1^]** | **Pd loading [wt%]** | **In loading [wt%]** |
| --- | --- | --- |
| 0.0027 | 1.4 | 0.3 |
| 0.0111 | 1.4 | 1.1 |
| 0.0222 | 1.3 | 2.0 |
| 0.0345 | 1.4 | 2.5 |
| 0.0444 | 1.3 | 3.6 |

### Grinding of PdIn/ZrO_2_ aerogels to produce reference samples

Reference materials were prepared by gently grinding 40 mg of the calcined PdIn/ZrO_2_ (1.3 wt% Pd, 1.2 wt% In) in a mortar. Grinding was performed either dry or in the presence of 5 mL of ethanol to generate catalysts with distinct morphologies but identical inorganic composition, referred to as “dry-ground aerogel” and “wet-ground aerogel,” respectively.

### Characterization

Zeta potential measurements were performed to assess the surface charge of ZrO_2_ nanoparticles in dispersion. Washed and dialyzed dispersions were diluted to 0.5 mg mL^-1^ in 0.01 M NaCl + 0.01 M HCl (pH 2). The pH was adjusted stepwise from 3 to 12 using aqueous NaOH, and aliquots were loaded into folded capillary cells (DTS1070, Malvern). Measurements were conducted on a Zetasizer Nano ZS (Malvern Instruments, UK), with each aliquot measured in triplicate to ensure reproducibility.

Thermogravimetric analysis (TGA) was carried out on a Mettler Toledo TGA/DSC 3+ Star system. Approximately 5–10 mg of ground Pd/ZrO_2_ aerogel (1.5 wt% Pd) were heated from 30 to 800 °C at a rate of 10 K min^-1^ under 50 mL min^-1^ airflow, and mass loss was recorded continuously.

X-ray fluorescence (XRF) was used to determine Pd content (**Table S3**). Ground aerogel samples were analyzed using a Rigaku ZSX Primus IV spectrometer equipped with a 4 kW Rh source and LiF(200), Ge, PET, and RX26 crystal detectors.

Nitrogen physisorption measurements were carried out on a Quantachrome Autosorb iQ at 77 K using ~20 mg of aerogel granules that were degassed at 100 °C for at least 24 h prior to analysis. Specific surface areas were determined using the Brunauer–Emmett–Teller (BET) method, and pore size distributions were obtained by nonlocal density functional theory (NLDFT) using a cylindrical silica pore model (**Table S3**).

X-ray photoelectron spectroscopy (XPS) measurements were performed using a PHI Genesis spectrometer (Physical Electronics/ULVAC-PHI) with monochromatic Al Kα radiation and charge neutralization. The PdIn/ZrO_2_ aerogel samples were gently ground to a fine powder, mounted on double-sided carbon tape, and pressed to obtain a flat surface. Survey spectra were acquired at a pass energy of 112 eV, and high-resolution spectra at 55 eV. Data were analyzed using CasaXPS, with charge referencing to the C 1s peak at 285 eV. A background subtraction was applied, and elemental compositions were determined using sensitivity factor corrections, assuming a homogeneous surface.Scanning transmission electron microscopy (STEM) was performed on a FEI Talos F200X microscope operated at 200 kV and equipped with a Super-X EDXS detector for elemental analysis. Aerogel samples were fragmented with a scalpel, dispersed in ethanol (1–10 mg mL^-1^), and drop-cast onto S166 lacey carbon grids.

Powder XRD patterns of Pd/ZrO_2_ (1.1 wt% Pd) samples were acquired using Cu Kα radiation (45 kV, 40 mA) on a PANalytical X’Pert Pro diffractometer. The aerogels were gently ground and placed on Si zero-background holders.

Digital images of Pd/ZrO_2_ aerogel spheres and aerogel fragments were acquired using a Leica S9i digital stereo microscope. The aerogel spheres were placed on a diffuse white backlight to visualize translucency and color. The aerogel fragments were placed on a weighing paper and only illuminated from above.

Reflectance spectroscopy measurements were performed using a deuterium/halogen light source (Ocean Optics DH-2000-BAL) coupled to an integrating sphere (Ocean Optics ISP-50-8-R-GT). Reflected light was detected with a UV–vis–NIR spectrometer (Ocean Optics USB2000+XR, 188–1033 nm). Diffuse and specular contributions to the total reflectance were deconvoluted by measuring once with a Spectralon specular inclusion insert and once with a light trap. To prevent sample loss through the 10 mm optical port, a glass slide was used as a base. Its reflectance was measured separately and subtracted from the sample spectra. Powder samples (20 mg) were spread and gently flattened in a 10.5 mm cylindrical holder, whereas intact aerogel spheres (20 mg) were measured without flattening to preserve the bulk aerogel structure, forming a monolayer of spheres in the holder. A black cap was placed over the sample to block stray light from entering through the interstitial voids.

Cooling curves of the aerogel spheres and ground aerogel reference samples were recorded using the same aluminum cup (10 mm diameter) as in the photothermal reactor. For each measurement, 13 ± 0.1 mg of sample was placed in the cup. The ground aerogel reference samples were lightly pressed to ensure uniform contact with the base of the cup, while the aerogel spheres were placed without compression. The cup was heated to 300°C on a hot plate and equilibrated for 10 min, then placed on an ice-bath-cooled steel block. Temperature decay was monitored using an infrared thermography camera (IRCAM Millenium 327k S/M, thermal sensitivity < 20 mK).

### Heatable photoreactor and illumination system

Photocatalytic reactions were conducted in a custom-built stainless-steel photoreactor (**Figure S1).** The reactor featured a cylindrical central chamber, sealed at the bottom with a 3 mm thick alumina disk held in place by a ring-shaped steel clamp. Catalyst granules were loaded into a perforated aluminum cup with a diameter of 10.2 mm positioned on a 1 mm thick glass-fiber mat to minimize heat loss to the reactor block. Illumination was introduced vertically through a CaF_2_ window mounted on the top face of the chamber.

Temperature control was provided by four cartridge heaters embedded in the reactor block and monitored by a thermocouple located near the reaction chamber. A second thermocouple protruding into the chamber measured the internal gas-phase temperature directly. Reaction gases were introduced through a dedicated inlet and conditioned depending on the operation mode. For catalyst activation and purely thermal catalysis experiments, the feed was preheated to match the reactor temperature. During photothermal operation, the inlet gas was cooled to 15 °C using a water-cooled heat sink to stabilize the operating temperature and compensate for gradual block heating under intense illumination.

In photothermal mode, the chamber thermocouple measured the temperature of the aluminum sample holder rather than the catalyst granules themselves. To directly monitor catalyst temperature, an infrared (IR) camera (IRCAM Millenium 327k S/M) was aligned with the CaF_2_ window at an oblique angle (~45° from vertical). IR emissivity and filter parameters were calibrated using reduced Pd/ZrO_2_ granules over the range 200–300 °C. The LED assembly was mounted vertically above the window to allow normal incidence illumination while leaving the IR camera’s optical path unobstructed.

High-intensity illumination was provided by a custom-built LED system (**Figure S2**). A 77 W white COB LED (SPHWHAHDNH23YZT3D4, Samsung Semiconductor, Inc.) was mounted on a water-cooled aluminum heat sink maintained at 15 °C to prevent overheating. The LED, cooling fan, and support frame were integrated into a custom 3D-printed housing made of polylactic acid (PLA). Two aspheric condenser lenses (ACL50832U, Thorlabs) were mounted in series: the first lens collected and collimated the emission, while the second focused the beam onto the reactor window. The optical output was calibrated with a broadband thermal power sensor (S405C, Thorlabs), achieving irradiances up to 4.8 W cm^-2^ (≈ 48 suns, AM 1.5G equivalent) at the sample surface.


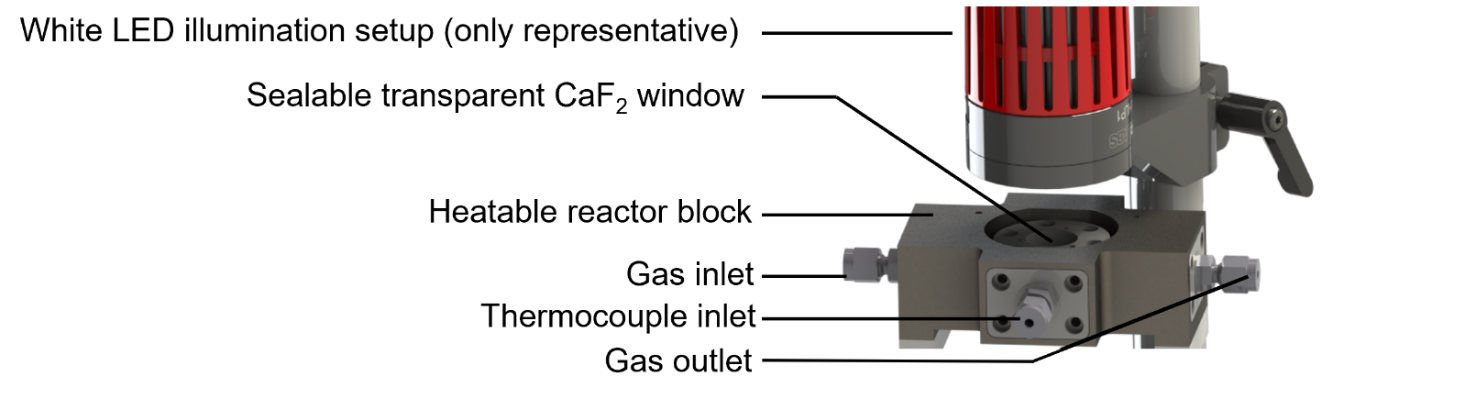


**Figure S1** | Digital rendering of the custom-built photoreactor. The reactor block is heated by embedded cartridge heaters and monitored by two thermocouples (block and chamber). The reaction chamber can be sealed with a CaF_2_ window, allowing both illumination and IR thermography. The LED light source is mounted vertically above the window, while the IR camera views the catalyst bed at an oblique angle.


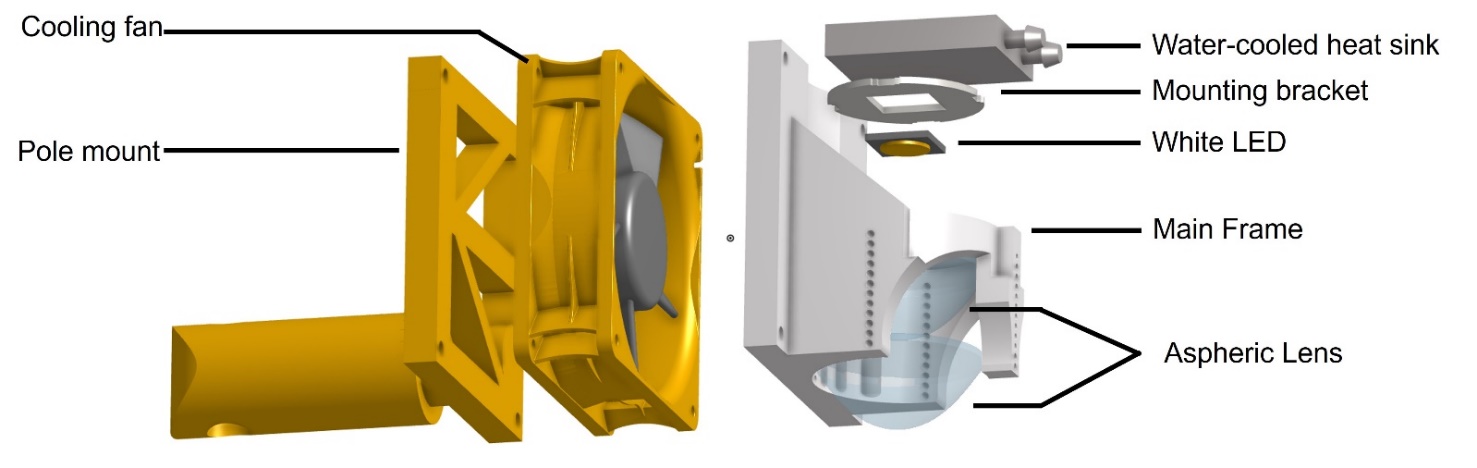


**Figure S2** | Digital rendering of the custom-built illumination assembly. The 77 W white COB LED is mounted on a water-cooled heat sink to maintain stable operating temperatures. The frame supports the cooling fan and pole mount, while two aspheric condenser lenses collect and focus the light onto the reactor window.

To directly monitor the temperature of individual catalyst granules during photothermal operation, an infrared (IR) camera (IRCAM Millenium 327k S/M) was used to capture thermographs of the aerogel bed under illumination. For quantitative analysis, the granules were collectively grouped into hand-drawn regions of interest (ROIs), and the average temperature across each ROI was calculated.

## Results and Discussion

### Surface charge and morphology of ZrO_2_ nanoparticles

To assess the surface charge of ZrO_2_ in the wet gel during impregnation, the zeta potential of the ZrO_2_ nanoparticle dispersion was measured (**Figure S3**). The surface charge of the nanoparticles is expected to largely translate to the gel structure, as gelation and assembly are not anticipated to significantly alter their acid–base properties. Upon adding base, the dispersions remained colloidally stable up to approximately pH 6, above which the suspensions became opaque and visible particle agglomeration occurred. Near the isoelectric point (pH ≈ 8), the dispersions were fully unstable. At pH 11, the absolute zeta potential was sufficiently high to prevent sedimentation, but the high transparency and small aggregate size observed under acidic conditions were not fully recovered, indicating that agglomeration upon crossing the isoelectric point is at least partially irreversible.

For impregnation, the pH was adjusted to control the surface charge of ZrO_2_ and maximize electrostatic adsorption of oppositely charged metal complexes. A strongly positive zeta potential is obtained near pH 5, which is suitable for deposition of EDTA-complexed metal ions, whereas a strongly negative potential is achieved near pH 11.

**Figure S3 |** Zeta potential of aqueous ZrO_2_ (3 nm) nanoparticle dispersions as a function of pH. Measurements were performed in 0.01 M NaCl + 0.01 M HCl at a solid concentration of 0.5 mg mL^-1^ with pH adjusted stepwise using aqueous NaOH.

To investigate the size and morphology of the ZrO_2_ building blocks used for the aerogels, an aqueous ZrO_2_ nanoparticle dispersion was diluted to 0.05 mg mL^-1^ in 0.01 M HCl and deposited onto S160 Cu TEM grids. STEM analysis revealed well-dispersed nanocrystals with an average diameter of ~2.5 nm, along with many agglomerates up to 20 nm (**Figure S4**). Whether this agglomeration originates from synthesis and washing steps or is induced during TEM sample preparation was not determined. High-resolution TEM imaging confirmed that all particles are highly crystalline, with lattice fringes clearly resolved.


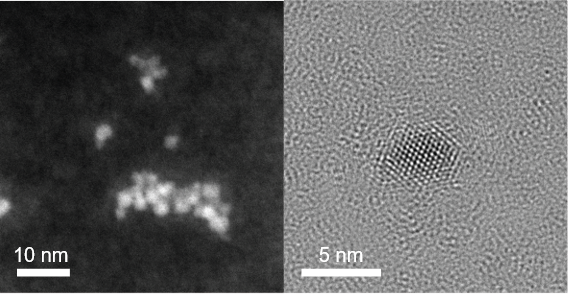


**Figure S4 |** HAADF-STEM image of ZrO_2_ nanoparticles (left) and TEM image of an individual nanocrystal (right). The nanoparticles are highly crystalline with well-resolved lattice fringes and an average diameter of ~2.5 nm.

### Light microscopy analysis of Pd/ZrO_2_ aerogel sphere size

The as-synthesized spheres exhibited a pale-yellow color arising from the impregnated Pd–EDTA complex (right, **Figure S5**) and had an average diameter of ~1.15 mm with a narrow size distribution (coefficient of variation, CV = 2.3%). Calcination in air at 300 °C led to a reduction in sphere diameter of ~10% (to ~1.0 mm) and a slight broadening of the size distribution (CV = 4.0%). Concomitantly, the spheres turned brown while remaining translucent (middle, **Figure S5**). This color change is suggested to reflect removal of the EDTA ligand and a corresponding change in Pd coordination from nitrogen- and carboxylate-based species to oxygen-dominated coordination on the ZrO2 surface. Carbonization of EDTA is unlikely to account for the observed coloration, as In/ZrO2 aerogels prepared under analogous conditions remain transparent before and after calcination. Following 18 h of photothermal catalysis, the spheres further contracted to an average diameter of ~0.9 mm and exhibited an increased size distribution (CV = 5.8%) (left, **Figures S5 and S6**). This broadening likely reflects nonuniform thermal conditions within the catalyst bed during operation. IR thermography confirmed that spheres located near the reactor wall were typically 20–30 °C cooler than those in the center, leading to spatially varying degrees of shrinkage.


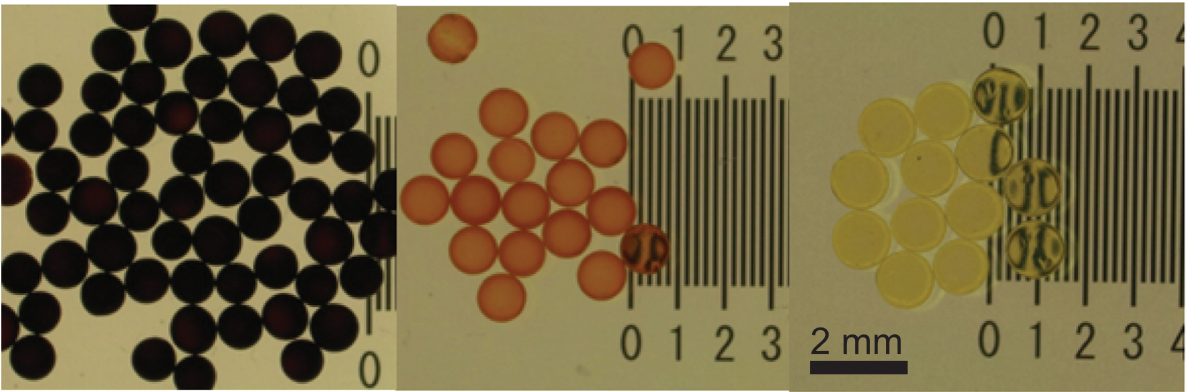


**Figure S5** | Light microscopy images of Pd/ZrO_2_ (1.5 wt% Pd) aerogel spheres after different treatments. (left) Spheres after 18 h of photothermal catalysis, appearing dark and nearly opaque. (middle) Calcined spheres (300 °C, air) showing a translucent brown appearance. (right) As-synthesized spheres with a pale yellow color.


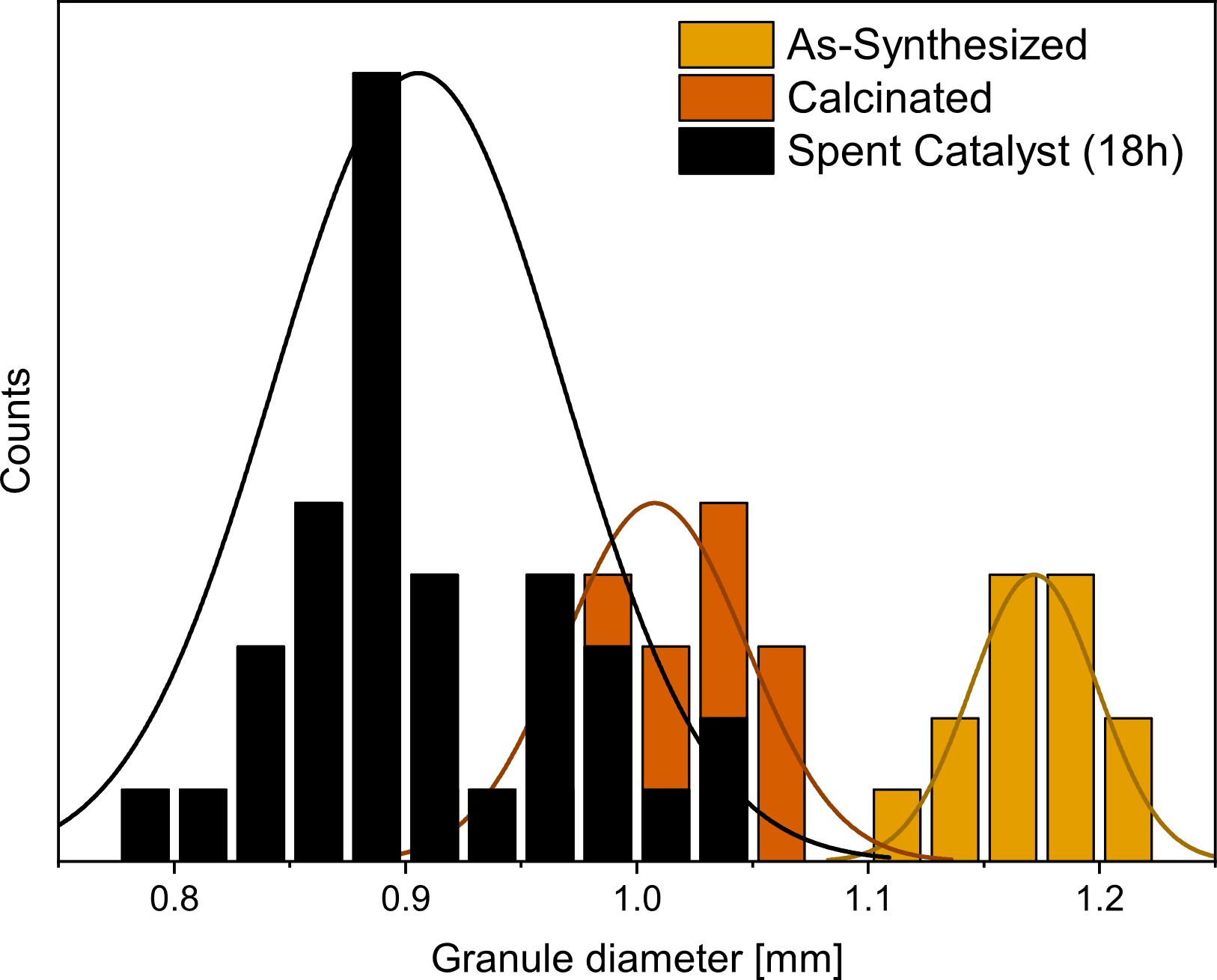


**Figure S6** | Size distribution of Pd/ZrO_2_ (1.5 wt% Pd) spheres after different treatments. The as-synthesized spheres display the narrowest size distribution and largest average diameter, whereas spent catalysts show the smallest average size and broadest distribution after 18 h on-stream.

### Macroscopic Pd distribution throughout the ZrO_2_ aerogel spheres

To assess the spatial distribution of Pd, as-synthesized Pd/ZrO_2_ spheres were fractured, and the fragments were examined using a Leica S9i digital stereo microscope. The yellow coloration indicates the presence of Pd–EDTA complexes, matching the color of the impregnation solution. An egg-shell–type distribution was observed, with Pd concentrated within ~100 µm of the outer surface (**Figure S7**). Calcined and H₂-reduced spheres (spent catalyst) exhibited the same egg-shell distribution.


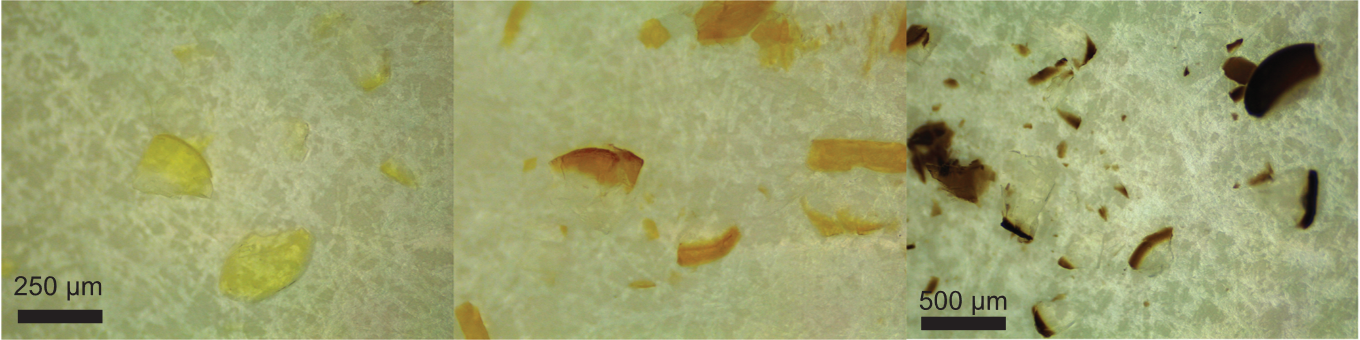


**Figure S7** | Light microscopy images of the (left) as-synthesized, (middle) calcined, and (right) spent Pd/ZrO_2_ (1.4 wt% Pd) aerogel show a consistent egg-shell–type Pd distribution, concentrated within the outer ~100 µm of the sphere surface.

### Thermal, compositional, and structural characterization of Pd/ZrO_2_ aerogels

Thermogravimetric analysis was used to assess the volatile content within the aerogels after different treatments (**Figure S8**). For the purposes of interpreting the TGA data, we assume that the surface water desorption and hydroxyl condensation behavior of zirconia follows trends reported for silica and TiO_2_.^1^ All samples exhibited a steep mass loss between 20 and 120 °C due to the desorption of physically adsorbed water, commonly present in samples stored under ambient conditions. Calcined and spent samples lost less free water, consistent with their reduced surface area and porosity (**Table S3**). Up to approximately 180 °C, additional mass loss is attributed to the desorption of surface-adsorbed water hydrogen-bonded to hydroxyls on the zirconia surface. At higher temperatures, up to about 500 °C, contributions from the condensation of surface hydroxyls are also observed.^2–4^ All samples show mass loss associated with both physisorbed water and hydroxyl condensation, but it is far more pronounced for the as-synthesized and calcined samples. The as-synthesized aerogel exhibited an additional, steeper mass loss between 200 and 300 °C, corresponding to the decomposition of residual EDTA overlapping with the water removal processes.^5^


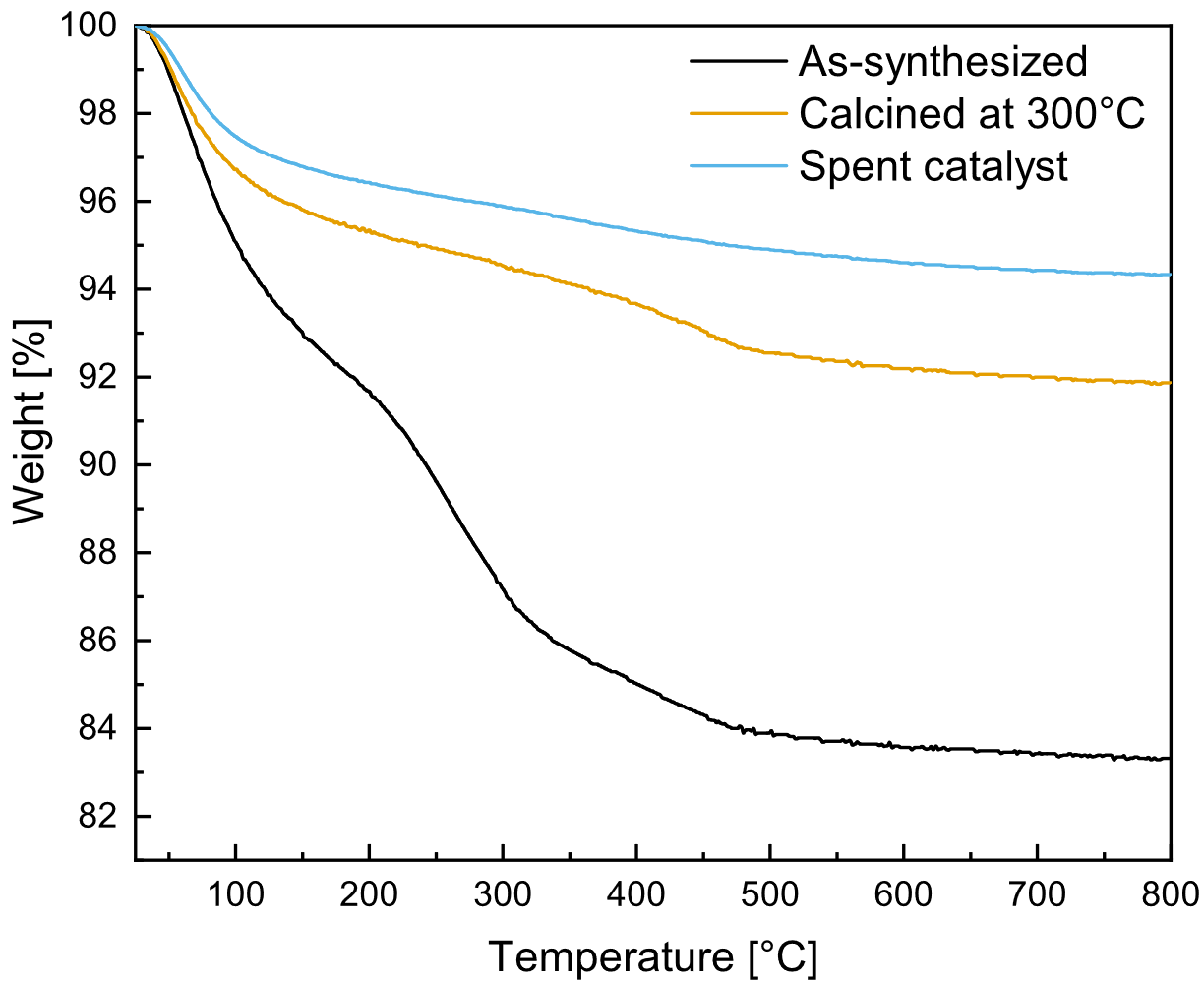


**Figure S8 |** TGA profiles of Pd/ZrO_2_ (1.5 wt% Pd) aerogel as-synthesized, calcined at 300°C in air, and the spent catalyst.

Full N_2_ sorption isotherms were measured for unloaded ZrO_2_ as well as Pd-loaded aerogels after calcination at 300 °C. The porosity of the non-impregnated, calcined ZrO_2_ sample was calculated as Φ = (V_p_ × ρ_s_) / [1 + (V_p_ × ρ_s_)], where the pore volume (V_p_) was obtained from the physisorption isotherm (V_p_ = 1.828 cm^3^ g^-1^) and the skeletal density (ρ_s_) was assumed to correspond to fully tetragonal ZrO_2_ (ρ_s_ = 6.09 g cm^-3^), resulting in a porosity of approximately 92%. The surface area of the calcined samples was independent of Pd loading, indicating that Pd remained highly dispersed and did not sinter during calcination at 300 °C (**Table S3**). After catalytic testing, however, the surface area decreased progressively with increasing Pd loading. In addition, the average mesopore diameter decreased by approximately 10% after reaction, independent of metal loading, consistent with partial sintering or densification of the ZrO_2_ backbone.

**Table S3 |** Palladium loadings (XRF), BET surface area, and average mesopore size of calcined (300 °C, air) and spent Pd/ZrO_2_ aerogel catalysts. Surface area decreases after catalysis, especially at higher Pd loadings, while mesopore size drops only ~10% independent of Pd content.

| **XRF** | **BET surface area** | | **Average mesopore size** | |
| --- | --- | --- | --- | --- |
| Pd loading [wt%] | Calcined at 300 °C [m^2^ g^-1^] | Spent catalyst [m^2^ g^-1^] | Calcined at 300 °C [nm] | Spent catalyst [nm] |
| 0.0 | 244 | na | 32 | na |
| 1.1 | 241 | 216 | - | 27 |
| 1.4 | 254 | 205 | 35 | 27 |
| 1.5 | 275 | 205 | 32 | 28 |
| 2.1 | 269 | 197 | 34 | 30 |
| 2.1 | 260 | 185 | 34 | 29 |
| 2.5 | 257 | 178 | 36 | 30 |

### Apparent activation energy of Pd/ZrO_2_ during thermal catalysis

The thermal catalytic activity of Pd/ZrO_2_ (1.4 wt% Pd) aerogels was evaluated in the absence of illumination. The catalyst was first reduced in 50 vol% H_2_/N_2_ at 300 °C for 1 h, after which the reaction gas feed was introduced. Product formation was monitored at temperatures between 180 and 300 °C, holding each temperature for 3 h. Arrhenius analysis of the measured rates yielded an apparent activation energy of 71.8 kJ mol⁻¹ for Pd/ZrO_2_, reflecting the thermocatalytic activity of the Pd sites (**Figure S9**).


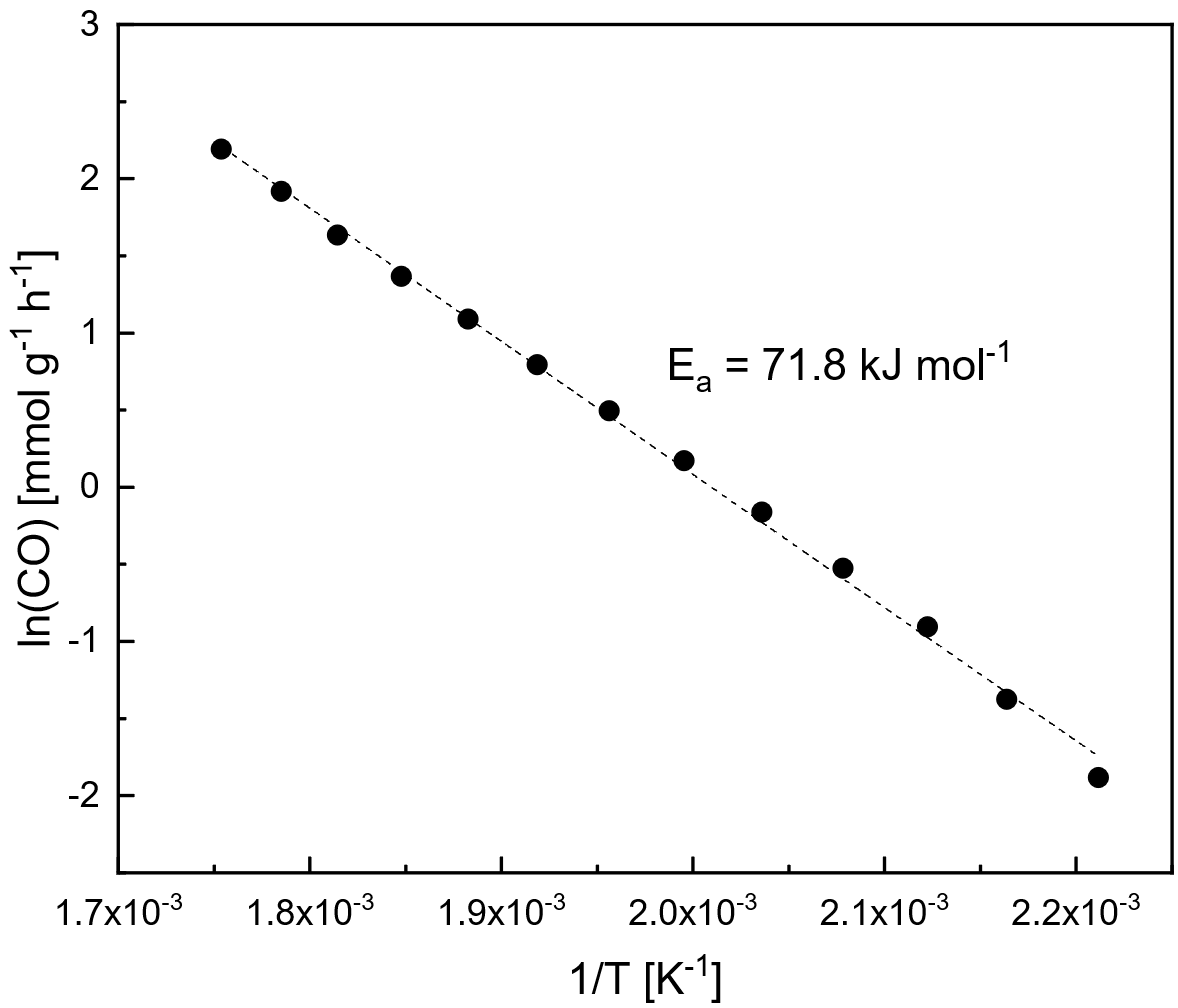


**Figure S9** | Arrhenius plot of CO space–time yield (STY) over Pd/ZrO_2_ (1.4 wt% Pd) aerogel catalysts under thermal conditions (no illumination). Apparent activation energy was obtained from the slope of ln(STY) versus 1/T. Reaction conditions: T = 180–300 °C; total pressure = 0.1 MPa; feed composition H_2_/CO_2_/N_2_ = 9:9:1 SCCM; gas hourly space velocity ≈ 3700 h^-1^; CO_2_ conversion ≈ 3%.

### Structural analysis of ZrO_2_ aerogel backbone in Pd/ZrO_2_ catalysts

To probe the influence of calcination on the aerogel framework, Pd/ZrO_2_ spheres were prepared for TEM analysis as described in the Methods section. HAADF-STEM images of the calcined material reveal an open, mesoporous network of interconnected ZrO_2_ nanoparticles (**Figure S10**). Particle size analysis gives an average diameter of ~3.5 nm, indicating modest particle growth during calcination. Much more pronounced structural changes are observed after 18 h of photothermal catalysis: the network of the spent catalyst is denser, the struts appear thicker and more coalesced, and the average particle diameter increases to ~5.8 nm (**Figure S11**). These results highlight the progressive sintering and coarsening of the ZrO_2_ backbone under reaction conditions.


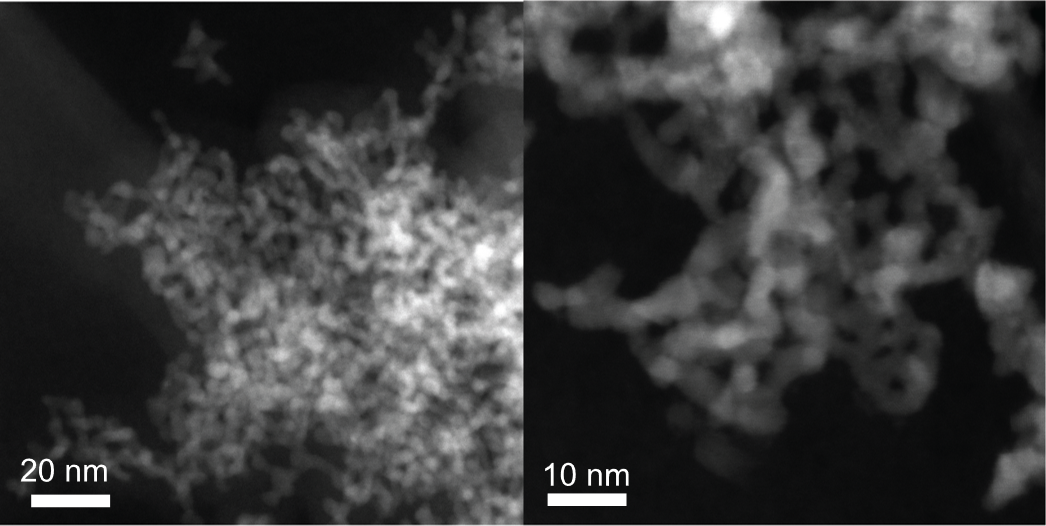


**Figure S10** | HAADF-STEM image of calcined Pd/ZrO_2_ (1.5 wt% Pd) aerogel showing an open, mesoporous network of interconnected ZrO_2_ nanoparticles (average diameter ~3.5 nm).


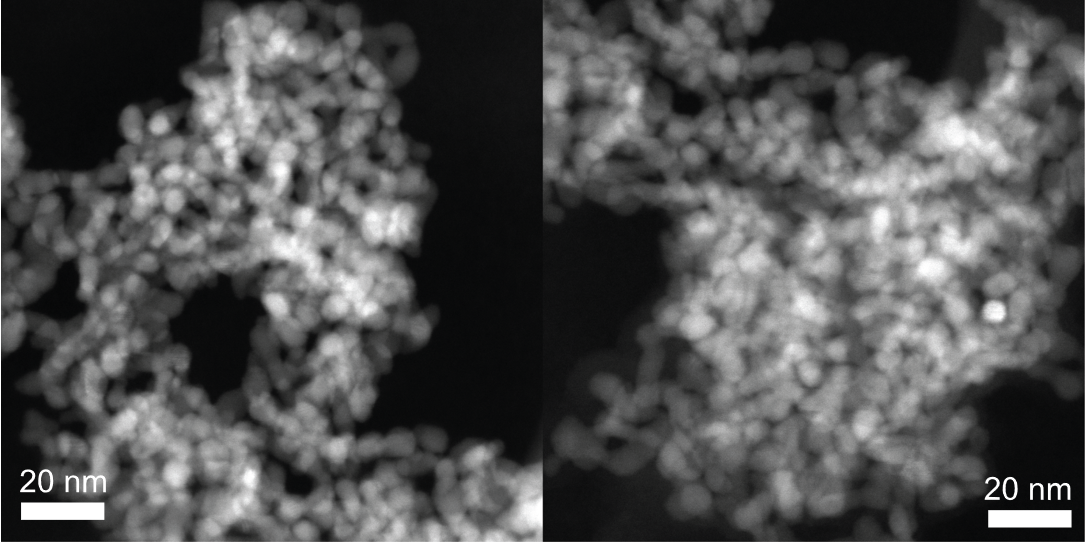


**Figure S11** | HAADF-STEM image of Pd/ZrO_2_ (1.5 wt% Pd) aerogel after 18 h of photothermal catalysis (spent catalyst), showing thicker, more coalesced ZrO_2_ struts and an increased average particle size (~5.8 nm).

The crystal structure of ground Pd/ZrO_2_ aerogel samples was characterized by powder X-ray diffraction (XRD) (**Figure S12**). Due to the small crystallite sizes, all diffraction peaks appear broad and no Pd reflections are observed. The as-synthesized aerogels exhibit a pattern characteristic of tetragonal ZrO_2_, in agreement with prior reports for similarly sized nanoparticles.^6^ After calcination, a shoulder at 28° and broad reflections between 40–50° emerge, indicative of partial transformation to the monoclinic phase. This monoclinic contribution increases slightly after catalysis, but the tetragonal reflections remain dominant. These observations are in line with size-dependent phase evolution of ZrO_2_, which transitions from tetragonal to monoclinic as crystallites grow.^6^


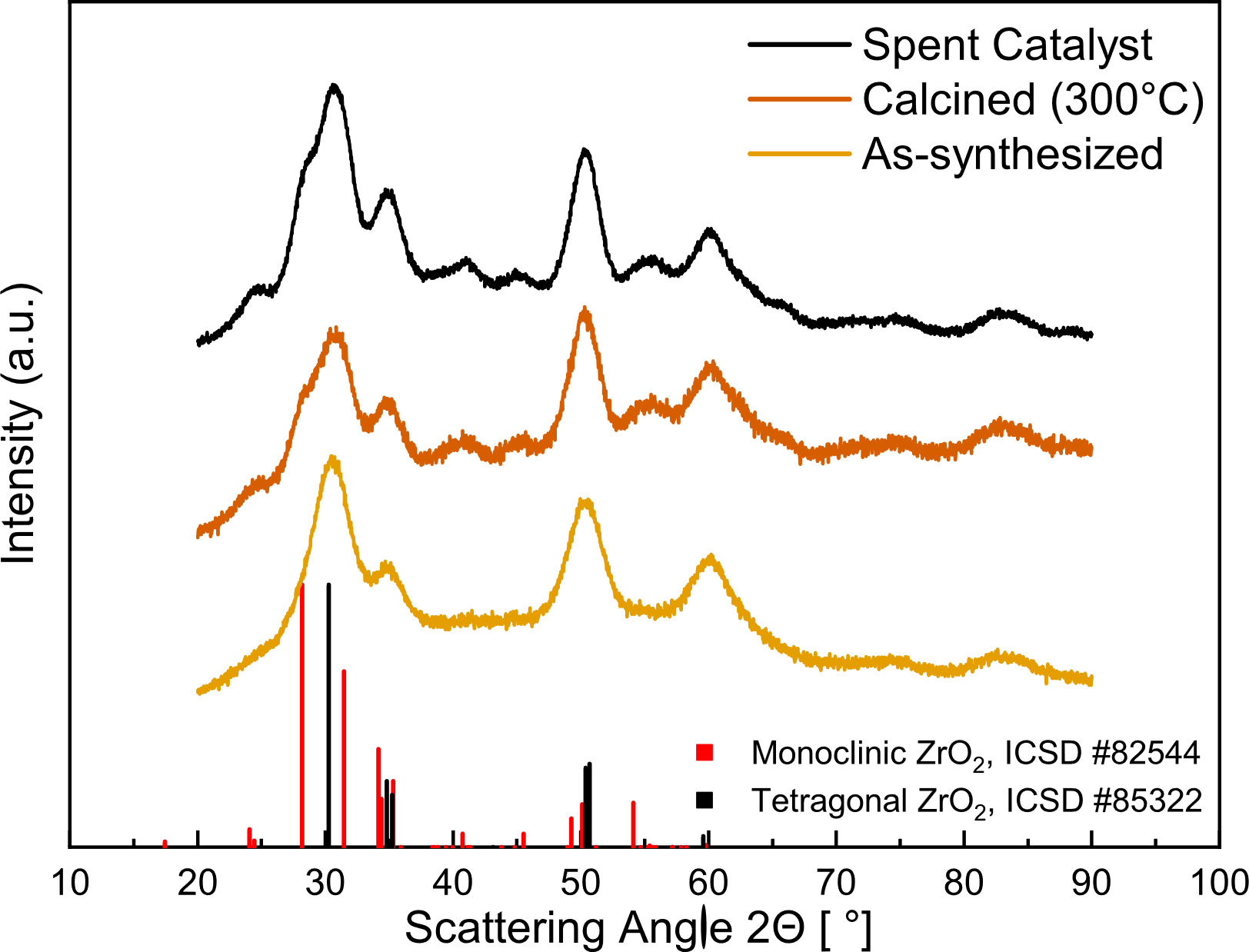


**Figure S12** | Powder XRD patterns of Pd/ZrO_2_ (1.1 wt% Pd) aerogels as-synthesized, after calcination, and after 18 h of photothermal catalysis. The tetragonal phase dominates initially, with a gradual increase in monoclinic features after thermal treatment and reaction.

### Pd dispersion on calcined Pd/ZrO_2_ sample

After synthesis and supercritical CO_2_ drying, the Pd/ZrO_2_ aerogel spheres were calcined in air at 300 °C for 24 h (1 °C min^-1^ ramp). TEM specimens were prepared as described in the Methods section. Fragments taken from the outer shell of the calcined spheres showed clear Pd–L signals in the energy-dispersive X-ray (EDX) spectra (not shown). **Figure S13** presents a representative high-angle annular dark-field scanning transmission electron microscopy (HAADF-STEM) image together with EDX maps for Zr and Pd. The Pd signal is weak, with some noise visible in the vacuum region that should appear completely dark. However, the Pd intensity is consistently higher on the ZrO_2_ surface, suggesting that Pd is present as highly dispersed species bonded to the ZrO_2_ framework. No Pd nanoparticles were observed anywhere in the STEM images.


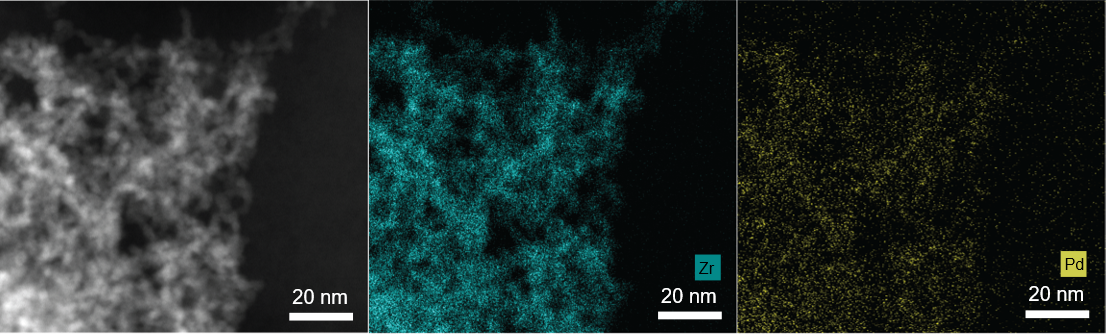


**Figure S13** | HAADF-STEM image and corresponding EDX maps of Pd/ZrO_2_ (1.5 wt% Pd) aerogel calcined in air at 300 °C. (Left) HAADF-STEM image of the aerogel fragment. (Middle) Zr EDX map. (Right) Pd EDX map showing a weak but spatially correlated Pd signal on the ZrO_2_ surface, indicating highly dispersed Pd species.

### Pd dispersion on reduced Pd/ZrO_2_ sample

Calcined Pd/ZrO_2_ (1.5 wt% Pd) aerogel spheres were placed in the reactor and heated to 200 °C under a 20 mL min^-1^ stream of 50 vol % H_2_ in N_2_. The temperature was held for 1 h to reduce the catalyst and then cooled to room temperature. TEM samples were prepared as described in the Methods section. For fragments taken from the outer shell of the catalyst spheres, Pd-L signals were clearly observed in the energy-dispersive X-ray (EDX) spectra (**Figure S14**). **Figure S15** shows representative HAADF-STEM images overlaid with Pd EDX maps, revealing the presence of well-dispersed Pd clusters with an average diameter of ~1.5 nm on the ZrO_2_ aerogel support.


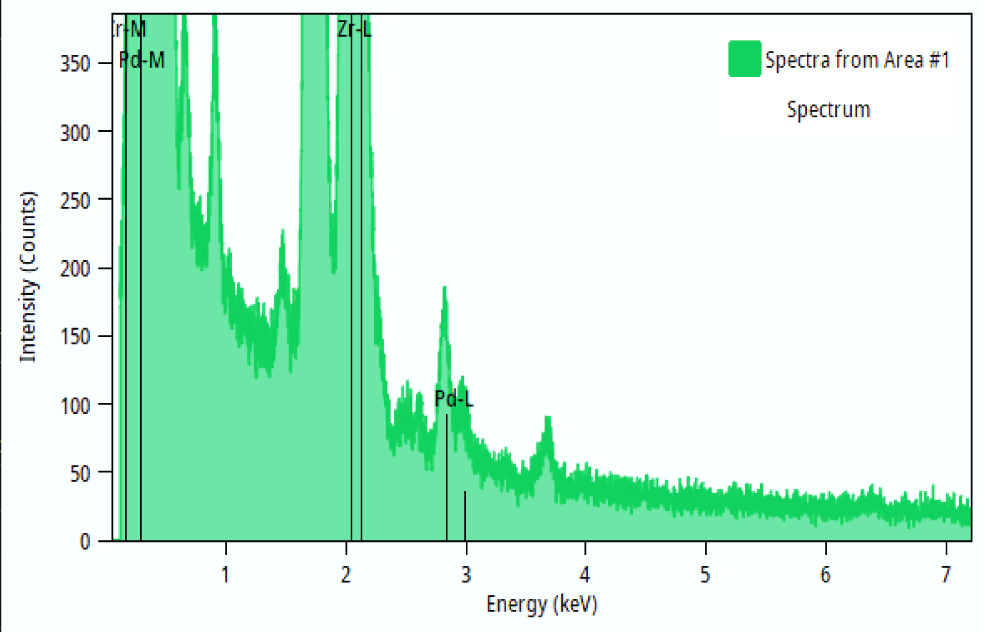


**Figure S14** | EDX spectrum acquired from a 40 × 40 nm region of a Pd/ZrO_2_ (1.5 wt% Pd) aerogel fragment, showing dominant Zr-L signals and clearly detectable Pd-L lines. Minor peaks originate from the Cu TEM grid and trace surface contaminants (e.g., Ca).


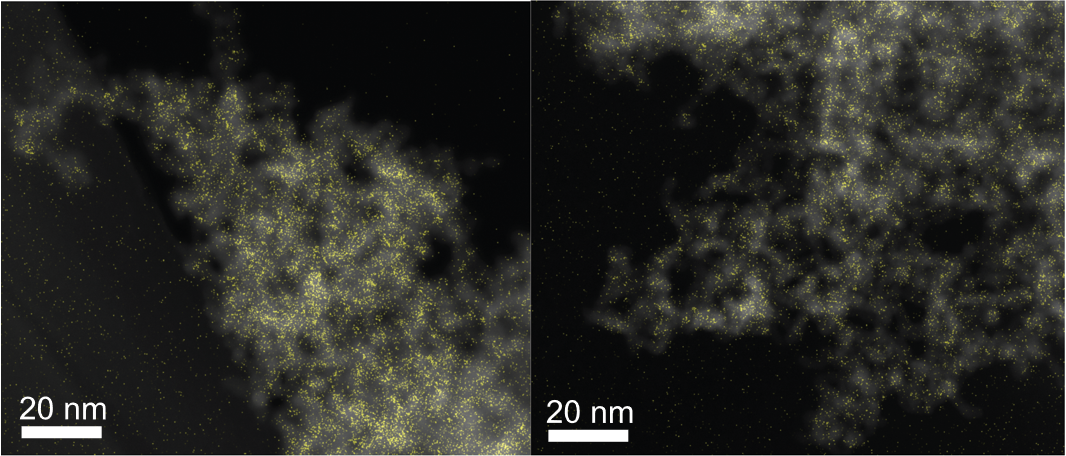


**Figure S15** | HAADF-STEM images overlaid with Pd EDX maps (yellow) of reduced Pd/ZrO_2_ (1.5 wt% Pd) aerogel fragments. Pd is present as small, well-dispersed clusters with an average size of ~1.5 nm.

### Pd dispersion on the spent photothermal Pd/ZrO_2_ catalyst

Pd/ZrO_2_ (1.5 wt% Pd) aerogel spheres were recovered from the photothermal reactor after 18 h on stream and analyzed by STEM and EDX mapping to evaluate Pd dispersion after prolonged catalysis. TEM samples were prepared as described in the Methods section. Fragments from the outer shell of the catalyst spheres exhibited more intense Pd-L signals in the EDX spectra compared to the calcined and fresh/reduced Pd/ZrO_2_ samples (not shown).

**Figure S16** shows representative HAADF-STEM images overlaid with Pd EDX maps, revealing Pd nanoparticles with an average diameter of ~3.0 nm on the ZrO_2_ support. The increase in particle size reduces the total Pd surface area, which likely contributes to the observed decrease in catalytic activity. Image analysis (ImageJ) of multiple regions was used to quantify the Pd particle size distribution. **Figure S17** compares the distributions for freshly reduced and spent Pd/ZrO_2_ catalysts, showing an increase in average particle size from 1.5 ± 0.5 nm to 3.0 ± 1.7 nm, along with a pronounced broadening of the distribution, indicating substantial sintering during reaction.


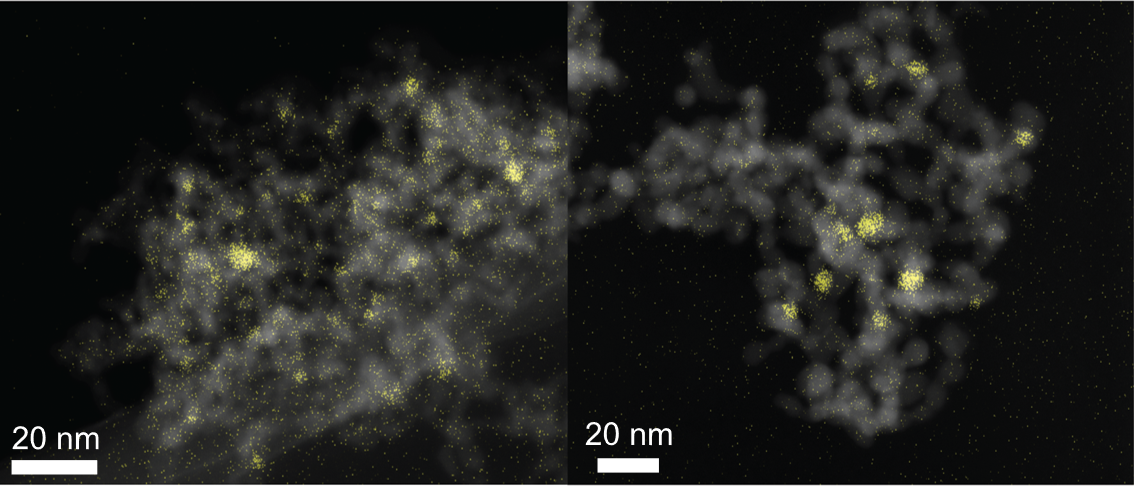


**Figure S16** **|** HAADF-STEM images of spent Pd/ZrO_2_ (1.5 wt% Pd) overlaid with Pd EDX maps, illustrating the growth of Pd nanoparticles on the ZrO_2_ aerogel after 18 h of photothermal catalysis.


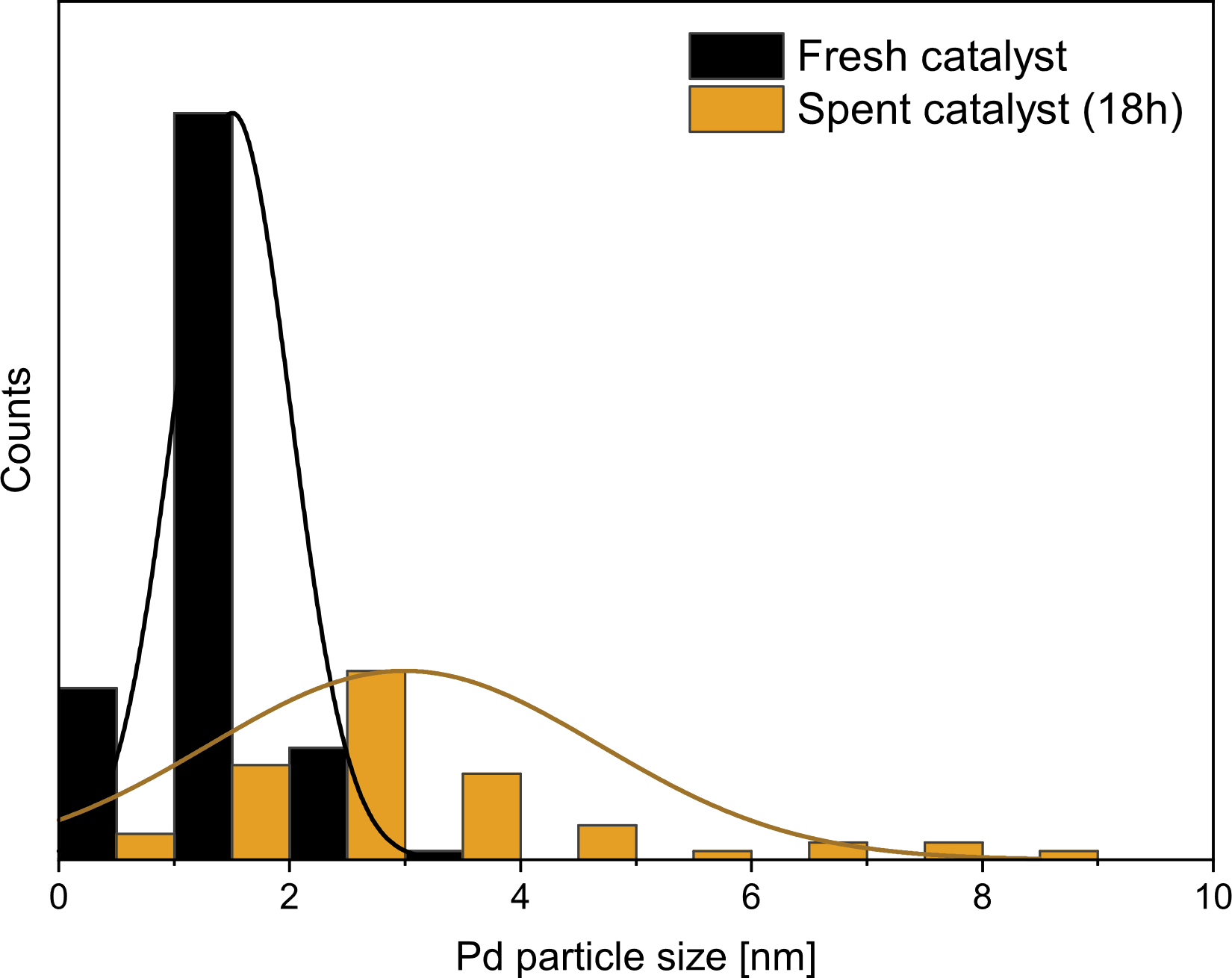


**Figure S17** | Pd nanoparticle size distributions on Pd/ZrO_2_ (1.5 wt% Pd) aerogel before and after photothermal catalysis, showing an increase in both average particle size and distribution width due to sintering.

### Compositional and structural characterization of PdIn/ZrO_2_ aerogel catalysts

The BET surface area of calcined PdIn/ZrO_2_ samples was independent of total metal loading (**Table S4**), indicating that Pd and In were highly dispersed and did not undergo significant sintering during calcination at 300 °C. After 18 h of photothermal catalysis, an average surface area loss of ~21 % was observed. For the bimetallic samples, the extent of surface area loss did not correlate with total metal loading (Pd + In) but followed the Pd loading, similar to the trend observed for monometallic Pd/ZrO_2_ catalysts. For example, samples containing 1.3 wt% Pd retained more surface area than those with 1.4 wt% Pd, suggesting that the Pd content plays a dominant role in sintering behavior, whereas indium remains well-dispersed on the ZrO_2_ support. As in the monometallic system, the average mesopore diameter decreased by ~13 % after catalysis, consistent with partial densification or sintering of the ZrO_2_ backbone.

**Table S4** **|** Active metal loadings (XRF), BET surface area, and average mesopore size of calcined (300 °C, air) and spent PdIn/ZrO_2_ aerogel catalysts.

| **XRF data** | | **BET surface area** | | **Average mesopore size** | |
| --- | --- | --- | --- | --- | --- |
| **Pd loading [wt%]** | **In loading [wt%]** | **Calcined at 300 °C [m^2^ g^-1^]** | **Spent catalyst [m^2^ g^-1^]** | **Calcined at 300 °C [nm]** | **Spent catalyst [nm]** |
| 1.4 | 0.3 | 251 | 182 | 33 | 29 |
| 1.4 | 1.1 | 267 | 199 | 33 | 28 |
| 1.3 | 1.2 | 271 | 221 | 33 | 30 |
| 1.4 | 2.0 | 239 | 174 | 33 | 29 |
| 1.3 | 2.5 | 255 | 218 | 33 | 27 |
| 1.3 | 3.6 | 262 | 231 | 34 | 30 |

To investigate the surface chemistry of the PdIn/ZrO_2_ aerogel, XPS measurements were conducted on samples in different states: as-synthesized (post supercritical drying), calcined (24 h, 300 °C, air), reduced (1 h, 200 °C, 1:9 H_2_:N_2_), and spent (18 h, photothermal catalysis). Zr, O, and C signals dominated the spectra and were readily quantified (**Table S5**). The surface Zr and O contents gave an atomic ratio of approximately 1:2, consistent with the expected stoichiometry of ZrO_2_. The carbon content was highest in the as-synthesized sample (approximately 3.4 wt%) and decreased to approximately 1.4 wt% in the other samples (**Table S5**). This indicates that residual organics, such as EDTA and solvents (acetone, dioxane), remained on the surface after supercritical drying but were largely removed upon calcination. The low carbon content in the calcined, reduced, and spent samples is typical for ambient air-exposed surfaces.^7^ The spent catalyst sample does not show more carbon content than the calcined sample, implying that coking is not responsible for catalyst deactivation. Indium signals were weaker but distinct, allowing quantification, with a content of approximately 1.2 wt%, in agreement with XRF measurements (**Table S5**). Nitrogen, detected only in the as-synthesized sample, is attributed to residual EDTA that is removed upon calcination. The Pd 3d signal overlapped with the much stronger Zr 3p peak, and other Pd peaks were too weak to resolve, preventing a reliable determination of Pd surface content or oxidation state by XPS. The survey spectra showing the peak positions for all elements are presented in **Figure S18**.

**Table S5** **|** Surface composition of the PdIn/ZrO_2_ aerogel determined by XPS for samples in different states.

| **Sample description** | **Zr [wt%]** | **In [wt%]** | **O [wt%]** | **C [wt%]** | **N [wt%]** |
| --- | --- | --- | --- | --- | --- |
| As-synthesized | 71.2 | 1.2 | 23.7 | 3.4 | 0.5 |
| Calcined (300 °C, air) | 73.4 | 1.4 | 23.8 | 1.3 | 0.0 |
| Reduced (200 °C, H_2_) | 73.1 | 1.4 | 24.1 | 1.4 | 0.0 |
| Spent catalyst | 73.4 | 1.4 | 23.8 | 1.4 | 0.0 |

_
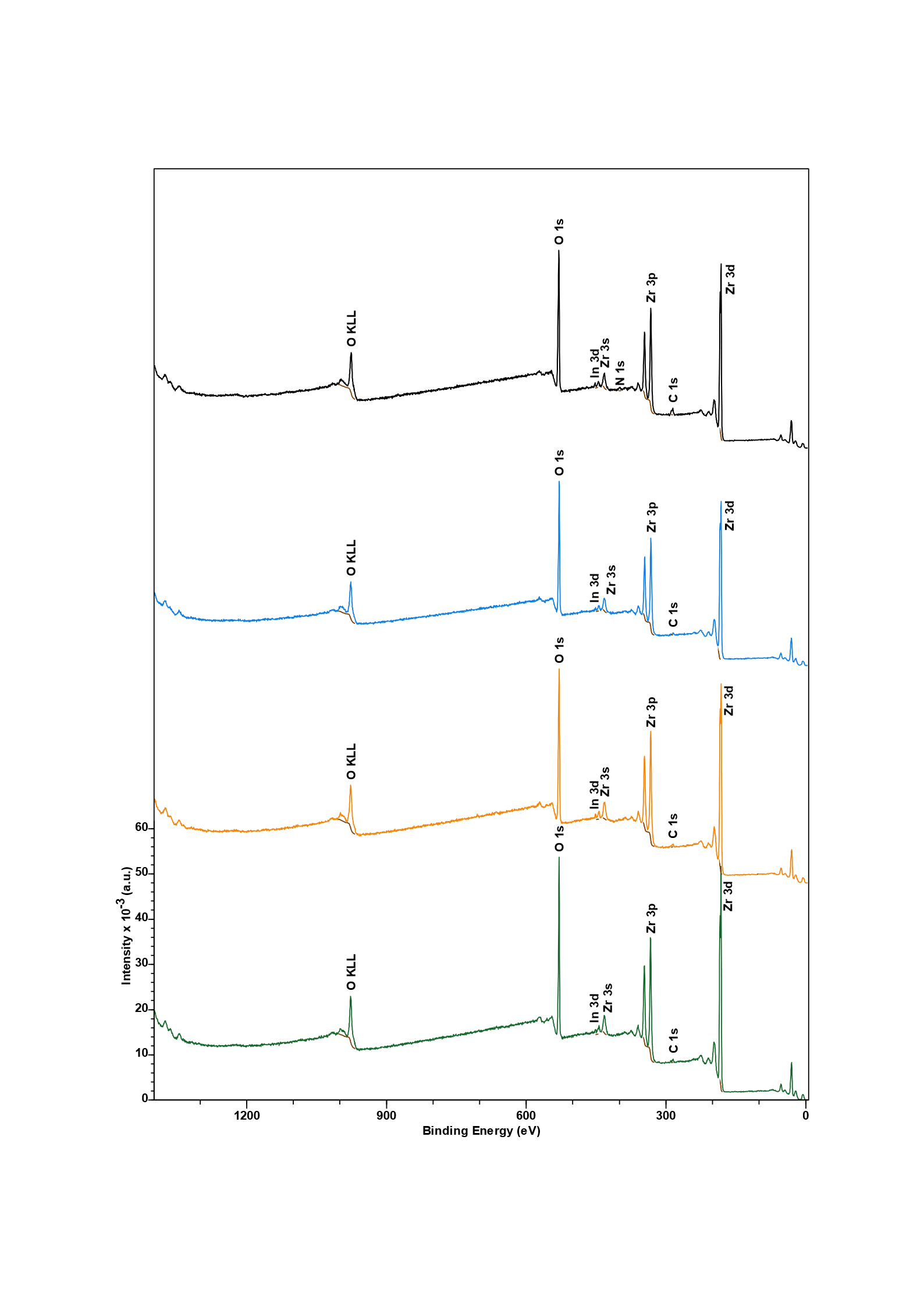
_

**Figure S18** | Survey XPS spectra of PdIn/ZrO_2_ aerogel in different states. All samples show signals corresponding to Zr, O, C, and In. Unlabelled features arise from additional orbitals of the same elements (e.g., O 2s at 23 eV).

### Apparent activation energy of PdIn/ZrO_2_ during thermal catalysis

The thermal catalytic activity of PdIn/ZrO_2_ aerogels was measured using the same procedure as for Pd/ZrO_2_. After activation in 50 vol% H_2_/N_2_ at 300 °C for 1 h, product formation was recorded at 180–300 °C with 3 h holds at each temperature. Arrhenius analysis yielded an apparent activation energy of 72.5 kJ mol^-1^, showing that the addition of In has only a minor effect on the activation barrier (**Figure S19**). This implies that the catalytic activity is largely dominated by Pd sites and that the co-location of In does not significantly alter their intrinsic activity.


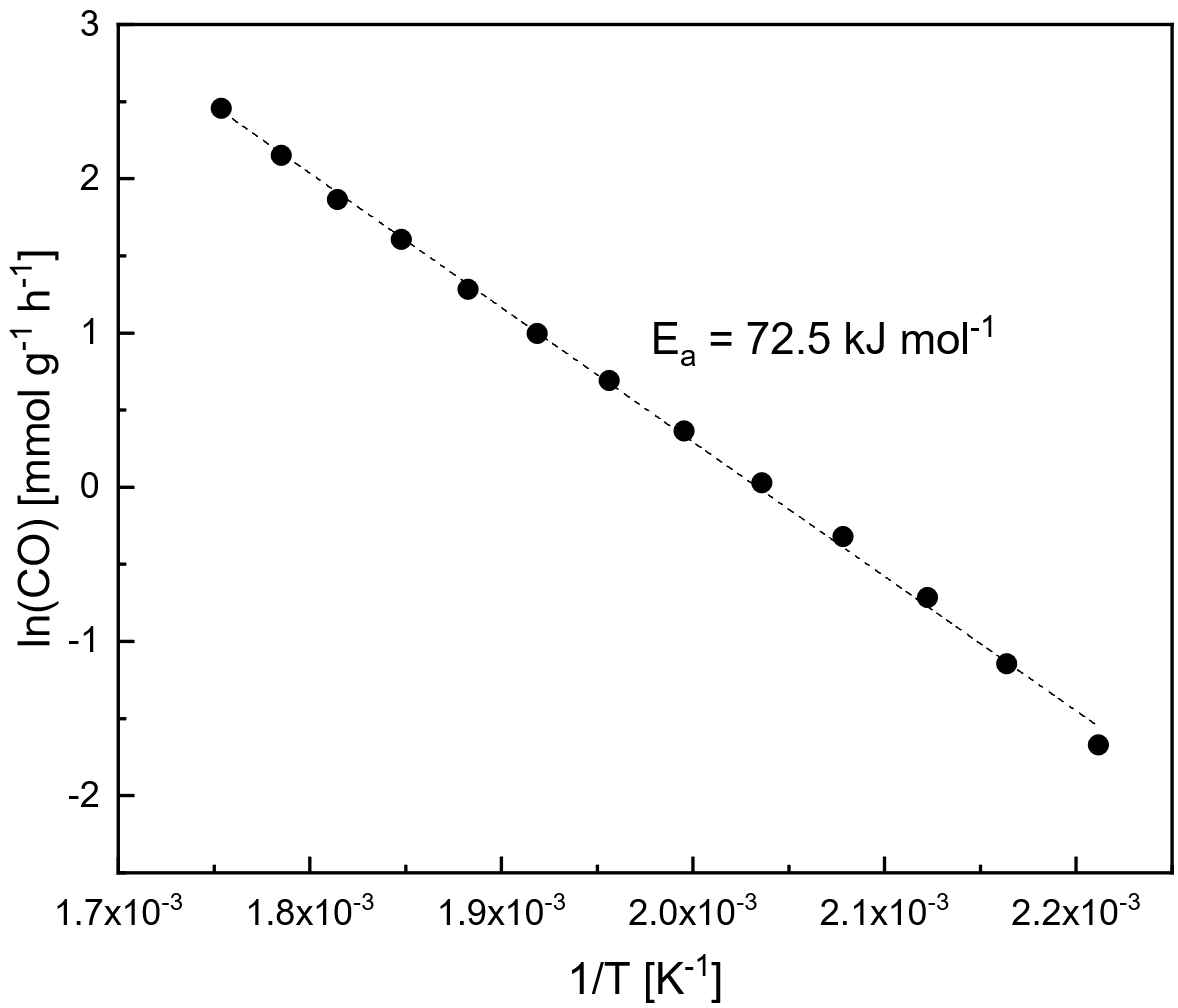


**Figure S19** | Arrhenius plot of CO space–time yield (STY) over PdIn/ZrO_2_ (1.4 wt% Pd, 1.3 wt% In) aerogel catalysts under thermal conditions (no illumination). Apparent activation energy was obtained from the slope of ln(STY) versus 1/T. Reaction conditions: T = 180–300 °C; total pressure = 0.1 MPa; feed composition H_2_/CO_2_/N_2_ = 9:9:1 SCCM; gas hourly space velocity ≈ 3700 h^-1^; CO_2_ conversion ≈ 3%.

Gas chromatograms during photothermal catalysis with PdIn/ZrO_2_

Gas chromatography (GC) was used to analyze the gas stream at the reactor outlet. After calibration with standard gas mixtures, the method enabled identification and quantification of the relevant gas-phase components. **Figures S20 and S21** show representative chromatograms obtained over PdIn/ZrO_2_ (1.4 wt% Pd, 1.2 wt% In). Column A (**Figure S20**) separates H_2_, N_2_, CH_4_, and CO. Column B (**Figure S21**) shows an initial combined signal from weakly retained species (H_2_, N_2_, CH_4_, and CO), followed by well-resolved CO_2_ and H_2_O peaks. No additional peaks were detected in either chromatogram. The same result was obtained for all other catalyst compositions investigated, including Pd/ZrO_2_ and PdIn/ZrO_2_ with varying indium content. While the formation of non-volatile species that are not transferred to the GC cannot be excluded, complementary analysis of the spent catalysts (e.g., XPS) did not provide evidence for the presence of such species.


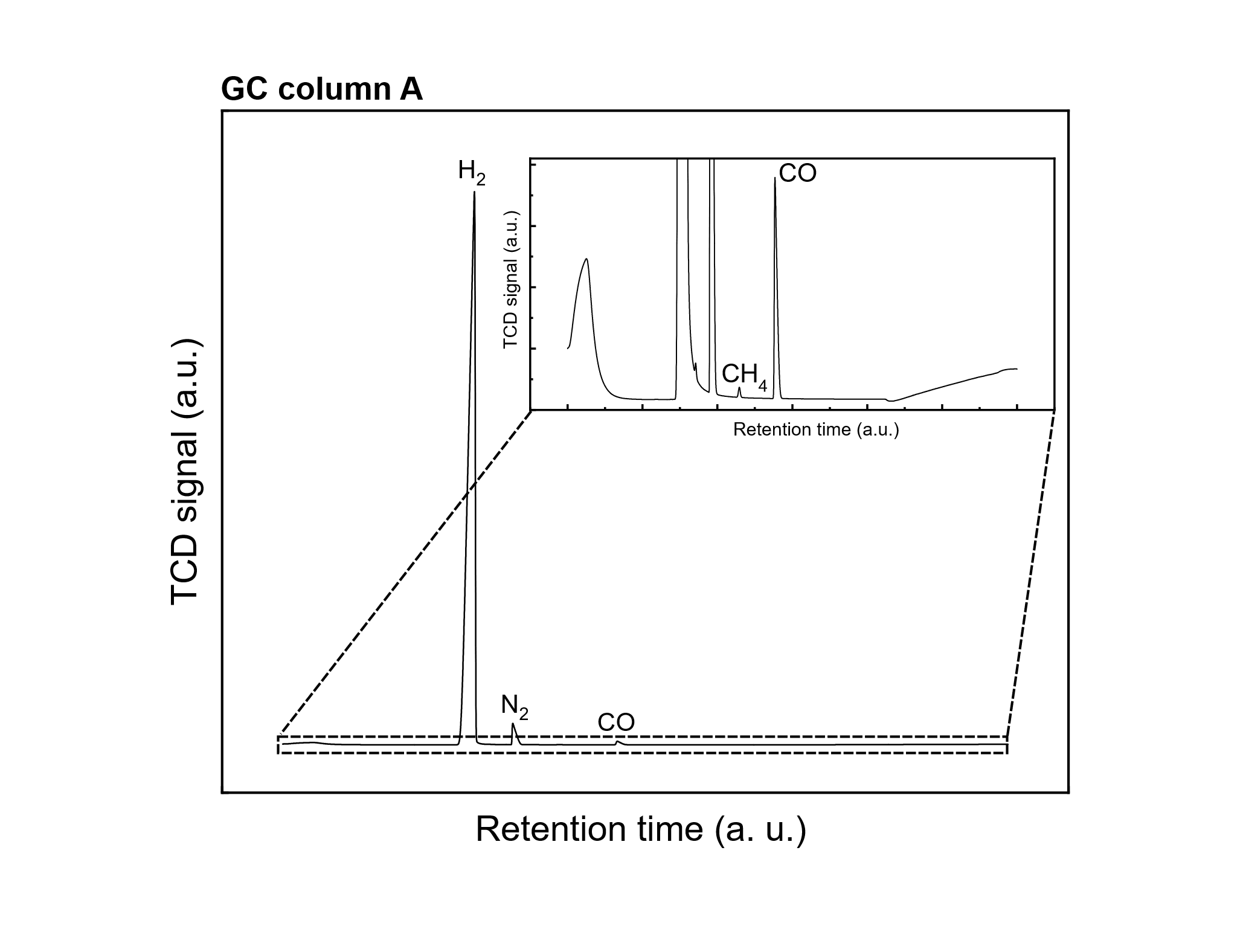


**Figure S20** | Gas chromatogram of column A obtained from the reactor outlet during photothermal catalysis over PdIn/ZrO_2_ (1.4 wt% Pd, 1.2 wt% In) at a time on stream of 2 h. The chromatogram shows a dominant H2 signal alongside weaker N_2_ and CO signals. The inset displays the same region at higher magnification, where the CH_4_ signal becomes visible. The rising baseline at longer retention times originates from the programmed increase in column temperature.


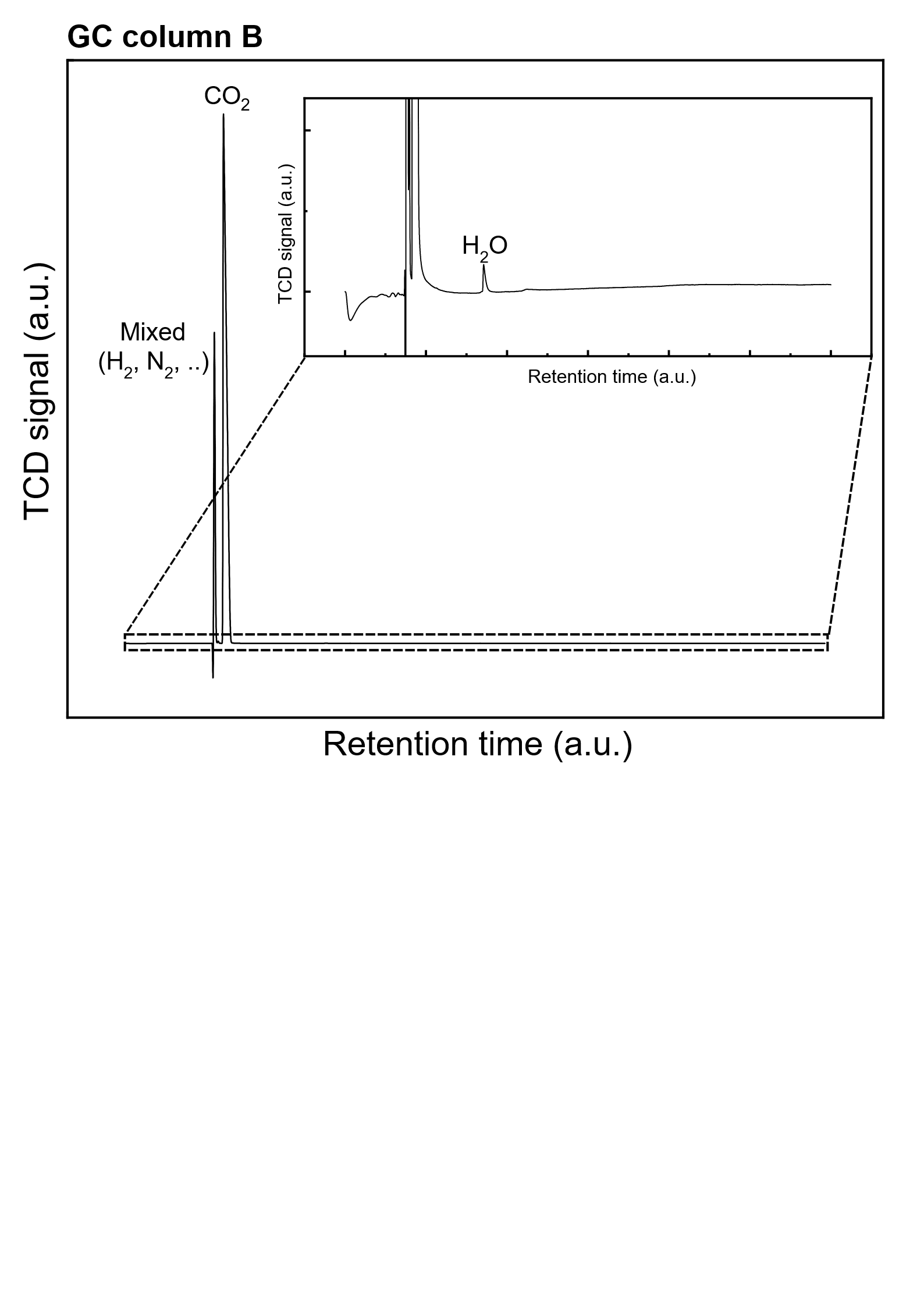


**Figure S21** | Gas chromatogram of column B obtained from the reactor outlet during photothermal catalysis over PdIn/ZrO_2_ (1.4 wt% Pd, 1.2 wt% In) at a time on stream of 2 h. The chromatogram shows a dominant signal from weakly retained species, followed immediately by the CO_2_ peak at slightly longer retention time. The inset displays the same region at higher magnification, where the H_2_O signal becomes visible at longer retention times.

### Infrared thermography of PdIn/ZrO_2_ aerogel spheres during photothermal catalysis

To directly monitor the temperature of individual catalyst granules during photothermal operation, an infrared (IR) camera (IRCAM Millenium 327k S/M) was used to capture thermographs of the aerogel bed under illumination. **Figure S22** shows a representative IR image of a fresh, reduced PdIn/ZrO_2_ catalyst under 4.8 W cm^-2^ white-light irradiation with a 20 sccm N_2_ flow. Catalyst bed temperatures ranged from ~270 to 330 °C.

Although the granules appear opaque under visible light, they are partially transparent in the mid-infrared spectrum used by the thermal camera. Consequently, the thermographs display patterns similar to those observed for transparent ZrO_2_ aerogel spheres under visible light, caused by refraction of IR light within their spherical bodies. Observed differences in average temperature between granules are primarily attributed to heat-loss variations: granules without direct neighbors or in contact with the metallic sample holder wall lose more heat and exhibit lower average temperatures. The illumination beam had a slight center-to-rim intensity gradient, but this is unlikely to explain the observed temperature variations, since colder granules were sometimes observed in the center of the bed.


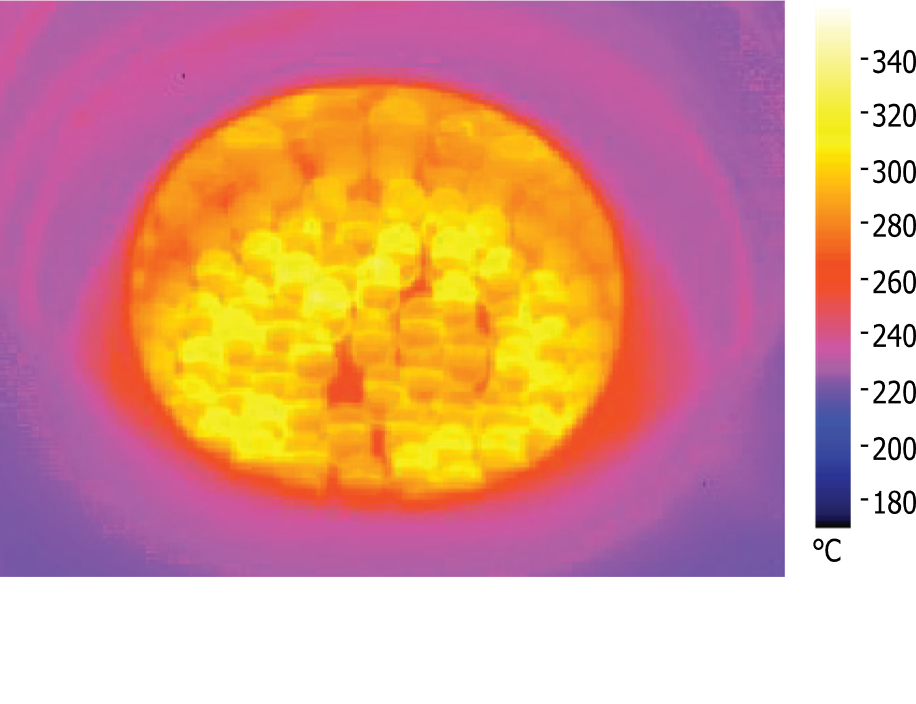


**Figure S22** | Representative IR thermograph of a monolayer of PdIn/ZrO_2_ (1.4 wt% Pd, 1.1 wt% In) aerogel spheres under 4.8 W cm^-2^ illumination with a 20 sccm N_2_ flow. Spheres in contact with neighbors generally exhibit higher temperatures, while those in contact with the metallic sample holder or isolated from neighbors are cooler. Mirror images of some spheres are visible on the aluminum walls of the sample holder due to IR reflection.

### Photothermal activity of PdIn/ZrO_2_ catalysts

PdIn/ZrO_2_ aerogel spheres with constant Pd content (1.3–1.4 wt%) and varying indium loadings (**Table S4**) were tested in the photothermal reactor. The initial CO space–time yield (STY) was plotted as a function of In content and compared with the corresponding average catalyst temperatures measured by IR thermography (**Figure S23**). Addition of a small amount of indium (0.3 wt%) slightly increased the initial STY but further increases in In loading did not improve activity. The catalyst temperature followed the same trend, indicating that the small STY increase is associated with slightly enhanced light absorption and modestly higher catalyst bed temperatures.

**
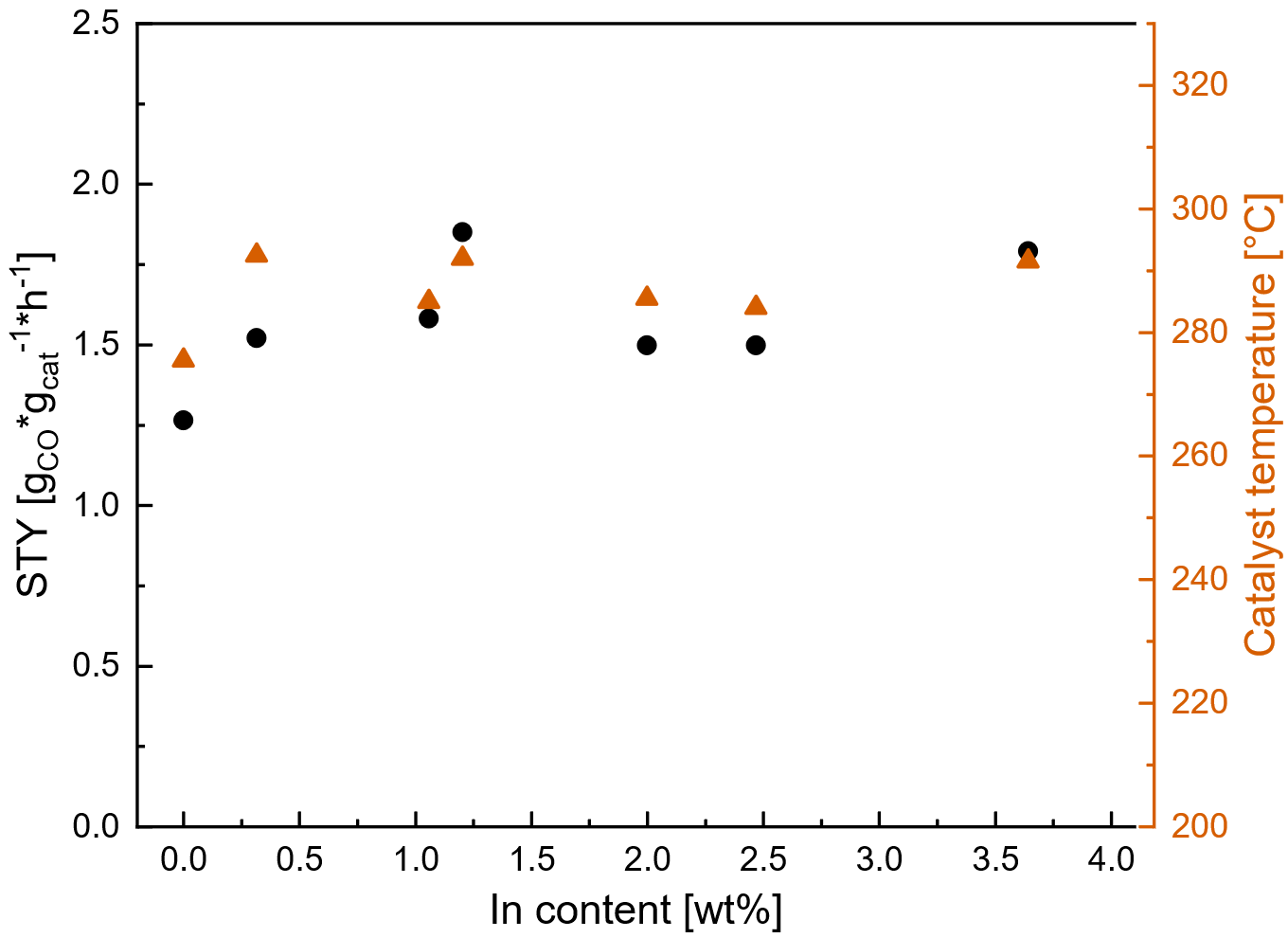
**

**Figure S23 |** Photothermal performance of PdIn/ZrO_2_ aerogel catalysts. Initial CO space–time yield (STY, left axis) of PdIn/ZrO_2_ spheres (~1.4 wt% Pd) as a function of In content. Corresponding average catalyst bed temperatures measured by IR thermography are shown on the right axis. Reaction conditions: light intensity, 4.8 W cm^-2^; total pressure, 0.1 MPa; feed composition, H_2_/CO_2_/N_2_ = 9:9:1 SCCM; gas hourly space velocity (GHSV), ~3700 h^-1^; CO_2_ conversion, ~5%.

### Long-term stability of the PdIn/ZrO_2_ catalyst in photothermal catalysis

PdIn/ZrO_2_ (1.6 wt% Pd, 1.2 wt% In) was selected for long-term stability testing, as it exhibited high CO STY at comparatively low metal loading, making it a more efficient composition. The pretreatment and photothermal reaction conditions were identical to those used in the shorter experiments, while the time-on-stream was extended to 110 h. **Figure S24** shows the evolution of the STY for the two main products, CO and CH_4_, as a function of time-on-stream. The catalyst exhibited a rapid initial deactivation over the first 7 h, during which the activity decreased to 55% of its initial value (corresponding to an average deactivation rate of 6.4% per hour). Following this initial period, the rate of deactivation decreased substantially and continued to decline with increasing time-on-stream. For example, between 60 and 100 h, the activity decreased by only 4.5% relative to the initial value (0.1% per hour). Although a fully stable plateau was not reached within the investigated timeframe, the deactivation rate after 110 h was very low. A small increase in CO STY observed after approximately 24 h is attributed to rearrangement of the catalyst granules within the reactor bed. Gradual sintering and the associated shrinkage of the aerogel can create space for rearrangement of the spheres. Such changes in packing can alter local light absorption and heat distribution, leading to slight variations in the average catalyst temperature and, consequently, the measured STY.


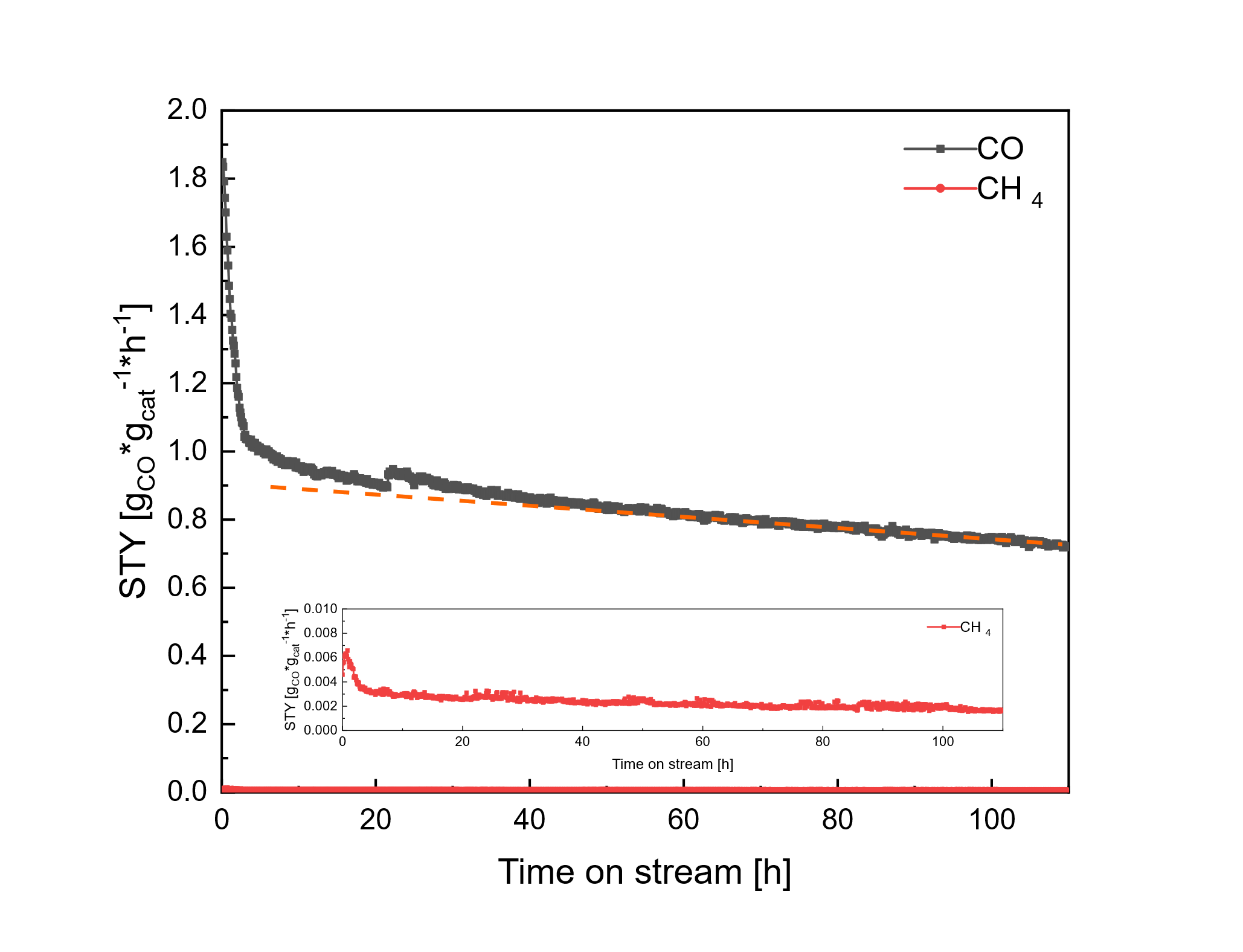


**Figure S24** | Space–time yield (STY) of CO and CH_4_ as a function of time on stream for a PdIn/ZrO_2_ (1.6 wt% Pd, 1.2 wt% In) aerogel under photothermal conditions. The catalyst shows an initial deactivation followed by a gradual approach to a more stable regime with a lower deactivation rate. The inset shows the corresponding CH_4_ STY, exhibiting a similar time-on-stream trend.

### Pd and In dispersion on the spent PdIn/ZrO_2_ catalyst

PdIn/ZrO_2_ aerogel spheres (1.4 wt % Pd, 1.1 wt % In) were recovered from the photothermal reactor after 18 h on stream and analyzed by STEM with EDX mapping to evaluate the dispersion of Pd and In after prolonged catalysis. TEM samples were prepared as described in the Methods section.

The EDX signal of In was very weak, consistent with a high degree of dispersion (**Figure** **S25**). Nonetheless, EDX elemental maps revealed partial co-location of In with Pd nanoparticles, suggesting intermixing of the two metals. Quantitative analysis (ImageJ) of Pd particle sizes from the Pd EDX maps yielded an average size of 3.5 ± 1.3 nm, comparable to that of the monometallic Pd/ZrO_2_ (1.4 wt% Pd) catalysts after reaction (3.0 ± 1.7 nm). These results indicate that the enhanced stability observed for certain PdIn/ZrO_2_ compositions is unlikely to originate from suppression of Pd sintering.


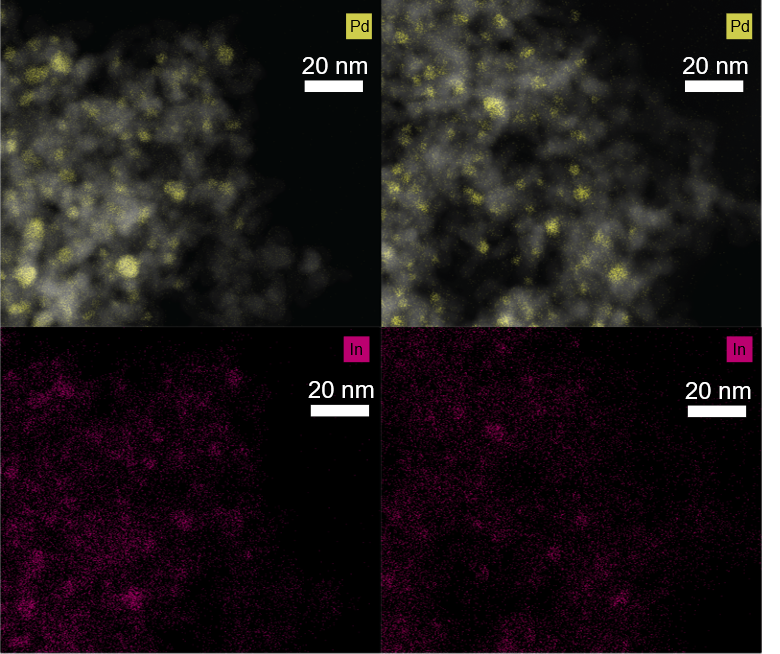


**Figure S25** | HAADF-STEM images of spent PdIn/ZrO_2_ (1.4 wt% Pd, 1.1 wt% In) overlaid with Pd EDX maps (top), showing Pd nanoparticles growth after 18 h of photothermal catalysis. EDX maps (bottom) reveal partial co-location of In and Pd nanoparticles.

### Optical, morphological and thermal properties of PdIn/ZrO_2_ aerogels compared to reference powders

PdIn/ZrO_2_ aerogel spheres and their dry- and wet-ground counterparts that had been tested in photothermal CO_2_ reduction for 18 h (spent catalysts) and subsequently stored under ambient conditions, were characterized for their optical properties. **Figure S26** shows the total reflectance spectra of spent PdIn/ZrO_2_, representing the sum of diffuse and specular components, for all three sample types. Intact aerogel spheres exhibited negligible reflectance, whereas dry-ground aerogel and wet-ground aerogel displayed average total reflectances of ~12 % and ~16 %, respectively. The linear increase in total reflectance with wavelength is attributed to the reduced absorption of longer-wavelength light by the Pd nanoparticles.^8^ This interpretation is supported by the data in **Figure S27**, which shows that the increase is more pronounced in the diffuse reflectance component than in the specular reflectance, consistent with absorption predominantly attenuating diffuse scattering.


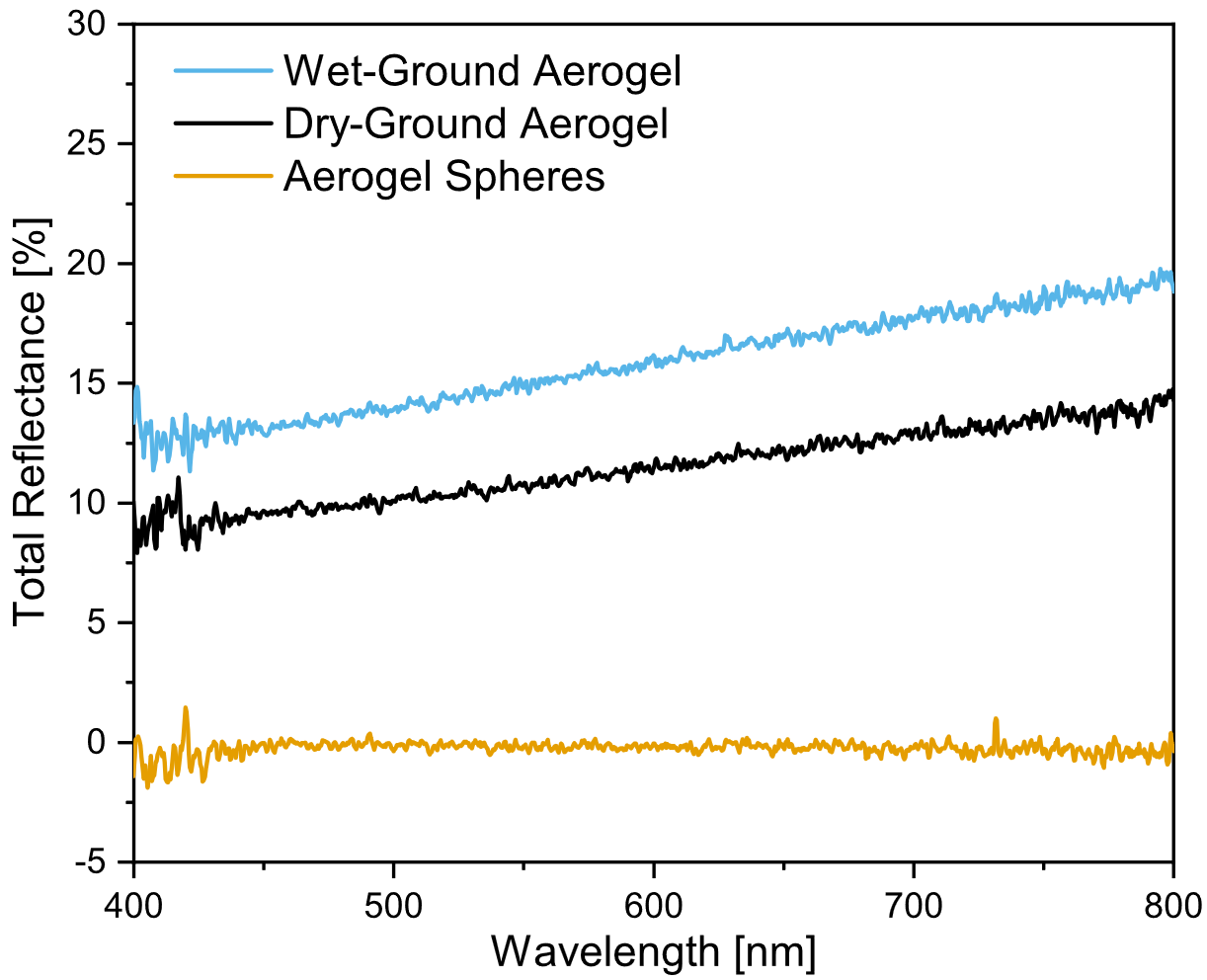


**Figure S26** | Total reflectance spectra of spent PdIn/ZrO_2_ (1.3 wt% Pd, 1.2 wt% In) catalysts in different forms: intact aerogel spheres, dry-ground aerogel (fragmented aerogel), and wet-ground aerogel (powder). Intact spheres exhibited negligible reflectance across the 200–800 nm range, whereas the ground aerogel and powder samples showed average total reflectances of ~12 % and ~16 %, respectively, indicating increased light scattering and reflection when the bulk aerogel is pulverized.


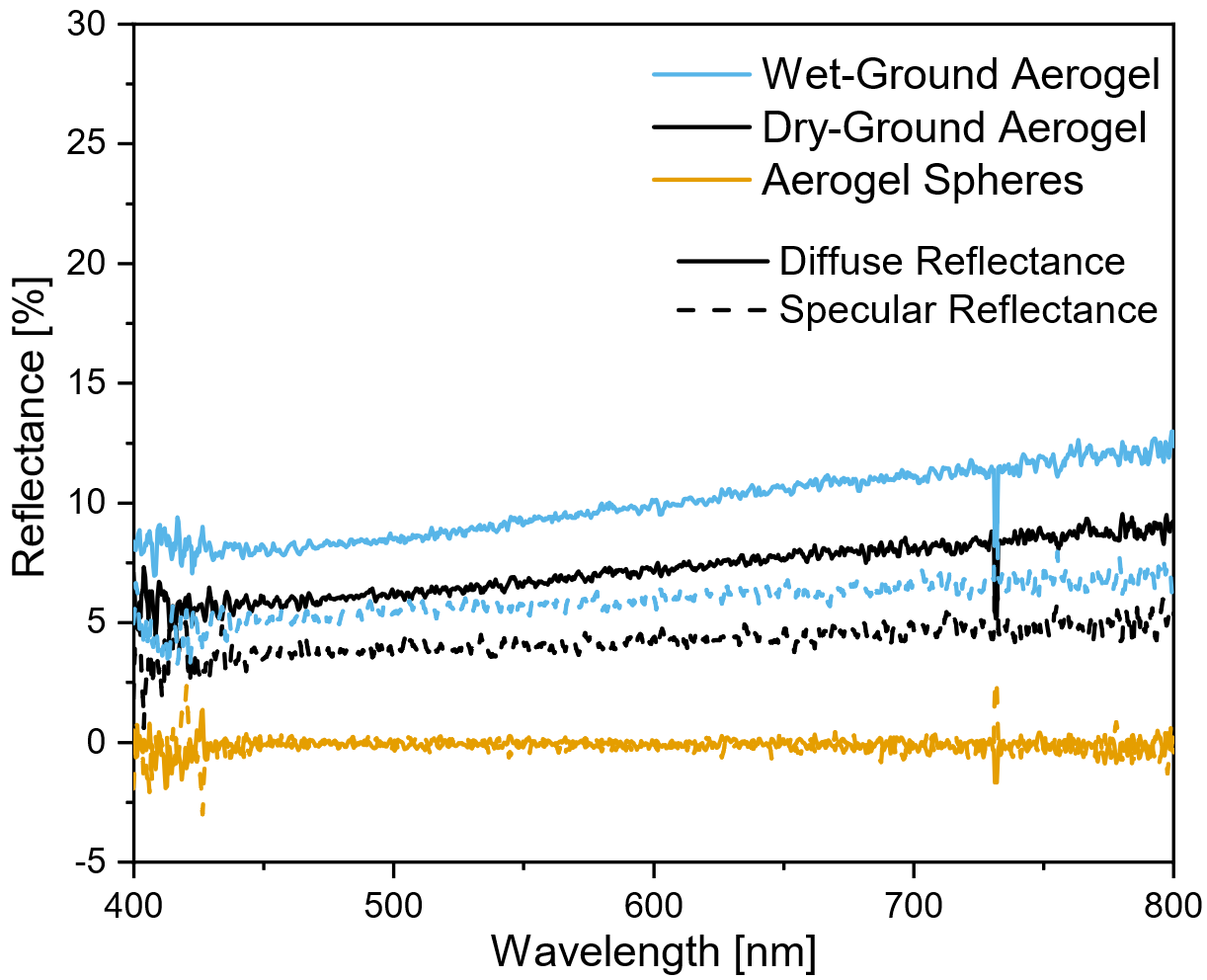


**Figure S27** | Diffuse and specular reflectance spectra of spent PdIn/ZrO_2_ (1.3 wt% Pd, 1.2 wt% In) catalysts in different forms: intact aerogel spheres, dry-ground aerogel (fragmented aerogel), and wet-ground aerogel (powder). Diffuse reflectance accounted for roughly twice the specular contribution, demonstrating that scattering is the dominant reflection mechanism. The stronger wavelength dependence of diffuse reflectance is consistent with preferential absorption of shorter-wavelength light by Pd nanoparticles.^7^

The surface area and pore structure of the fresh (calcined) and spent PdIn/ZrO_2_ aerogel spheres and the corresponding reference samples were characterized by N_2_ physisorption as described in the Methods section (**Table S6**). Dry grinding of the aerogel caused a modest reduction in BET surface area, likely because some nanoparticles detached from the aerogel framework and agglomerated. A more pronounced decrease was observed for the wet-ground aerogel, where the presence of ethanol during grinding induced structural collapse through capillary forces, leading to denser nanoparticle packing. After photothermal catalysis, all samples exhibited comparable BET surface areas, with the wet-ground aerogel retaining the highest final value. This likely reflects its lower reaction temperature (approximately 44 °C below that of the intact spheres), which mitigated sintering relative to the other samples.

The pore size distributions of the intact aerogel spheres and the dry-ground aerogel were both centered around ~33 nm, although the spheres exhibited a substantially higher mesopore volume (**Figure S28**). This suggests that mesopores serve as the primary crack initiation sites during grinding. Following fracture, many of these mesopores become open to the particle surface and are no longer detected by NLDFT analysis. In contrast, the wet-ground aerogel showed a distinctly different pore structure, with mesopores centered near ~12 nm. This shift is consistent with the complete collapse of the aerogel framework during wet grinding, resulting in a morphology that more closely resembles that of a conventional nanopowder catalyst.

**Table S6** **|** BET surface areas of fresh (calcined) and spent PdIn/ZrO_2_ (1.3 wt % Pd, 1.2 wt % In) catalysts. Intact aerogel spheres initially had the highest surface area but experienced the most significant loss during catalysis, resulting in similar surface areas across all spent samples.

| **Catalyst type** | **BET Fresh catalyst [m^2^ g^-1^]** | **BET Spent catalyst [m^2^ g^-1^]** |
| --- | --- | --- |
| Aerogel spheres | 271 | 221 |
| Dry-ground aerogel | 262 | 219 |
| Wet-ground aerogel | 243 | 229 |


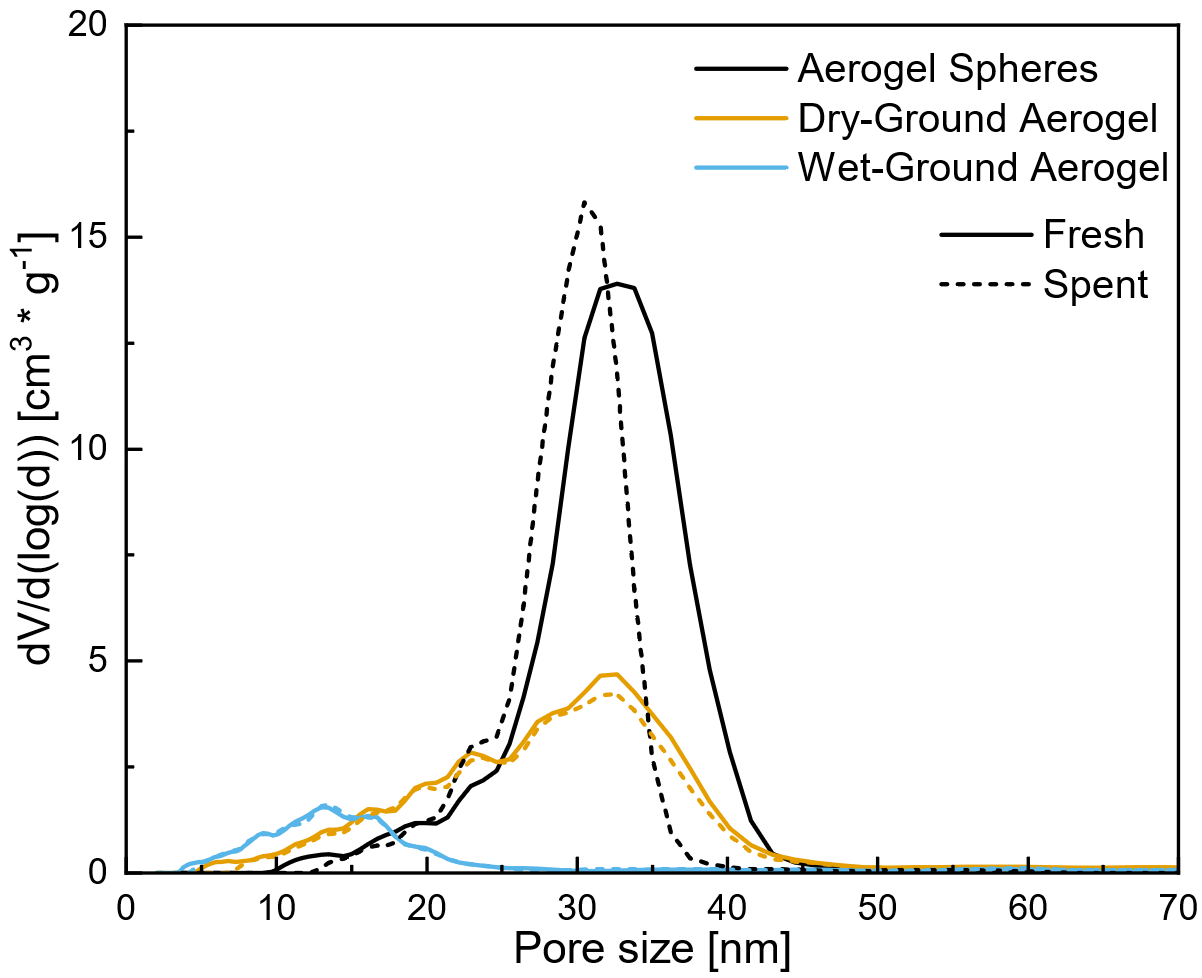


**Figure S28** | Pore size distributions of fresh and spent PdIn/ZrO_2_ (1.3 wt% Pd, 1.2 wt% In) catalysts in different forms: intact aerogel spheres, dry-ground aerogel (fragmented aerogel), and wet-ground aerogel (powder). Aerogel spheres and dry-ground aerogel exhibit similar mesopore distributions centered at ~33 nm, whereas the wet-ground sample shows a narrower distribution centered at ~12 nm, consistent with collapse of the aerogel network during wet grinding.

To investigate differences in effective thermal conductivity, cooling experiments were conducted on aerogel spheres (fresh and spent after 18 h and 110 h on stream) and ground reference samples (spent after 18 h on stream). All samples were heated in an aluminum cup, equilibrated at 300°C for 10 min on a hot plate, and then transferred to an ice-cooled steel block. Temperature decay was monitored using time-resolved IR thermography, and the average sample temperature over time is shown in **Figure S29**. The wet-ground aerogel exhibited the fastest cooling, followed by the dry-ground sample, while all aerogel sphere samples cooled more slowly. Notably, both spent aerogel spheres cooled faster than the fresh aerogel spheres. This trend aligns with BET surface area measurements and indicates that denser samples (i.e., ground references) possess higher effective thermal conductivity, enabling more rapid heat transfer to the aluminum cup and steel block. Temperature evolution was evaluated in the 200–300°C range, as the accuracy of the IR camera decreases below 200°C under the applied measurement conditions. Minor fluctuations around 230 and 210°C in the cooling curves are attributed to measurement artifacts and do not reflect physical behavior.


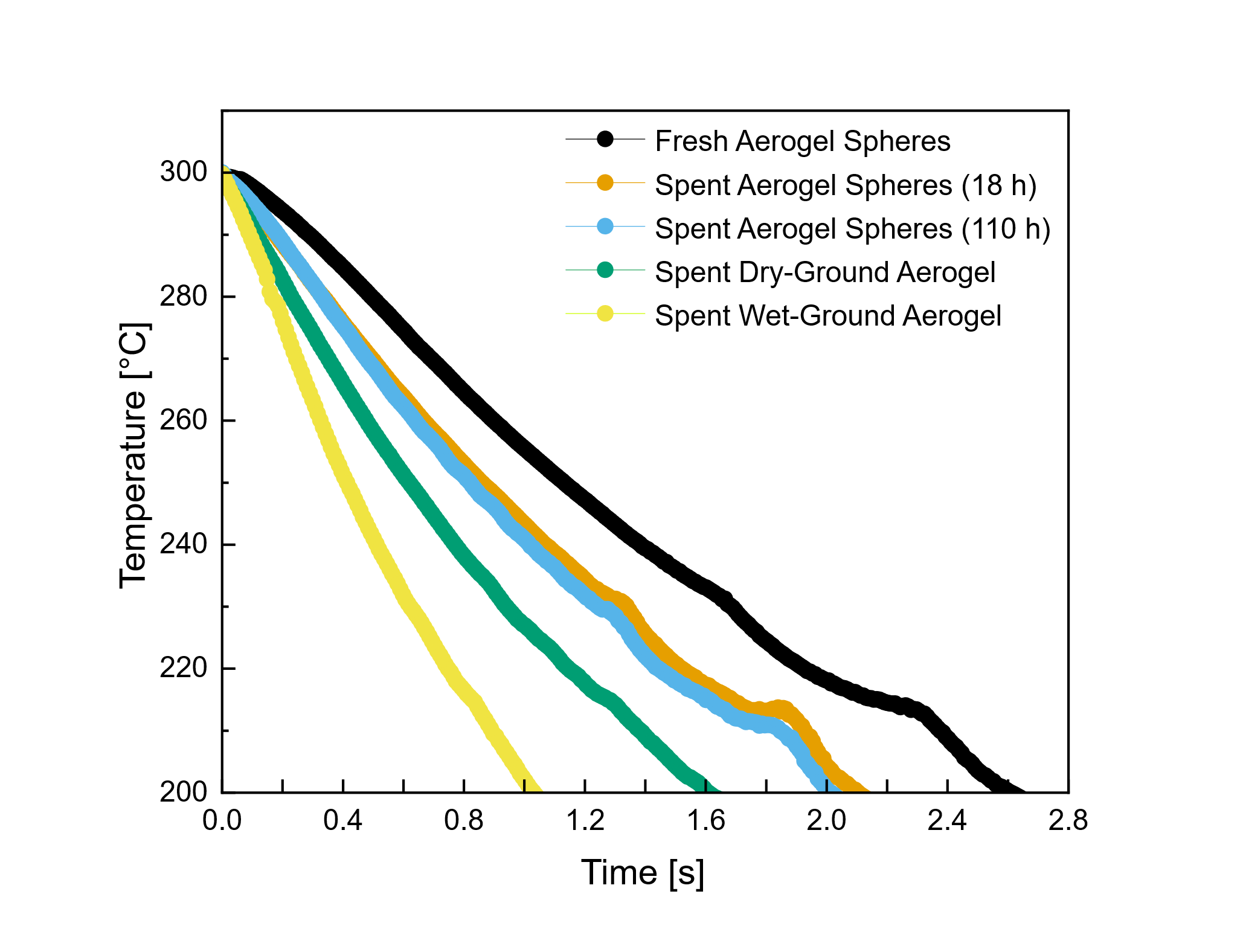


**Figure S29 |** Cooling curves of PdIn/ZrO_2_ (1.3 wt% Pd, 1.2 wt% In) catalysts in different structural forms: fresh and spent aerogel spheres, spent dry-ground aerogel (fragmented), and spent wet-ground aerogel (powder). The wet-ground aerogel exhibits the fastest cooling rate between 300 and 200 °C, while the fresh aerogel spheres cool most slowly, with the remaining samples showing intermediate behavior.

## References

(1) Pavan, C.; Santalucia, R.; Escolano-Casado, G.; Ugliengo, P.; Mino, L.; Turci, F. Physico-Chemical Approaches to Investigate Surface Hydroxyls as Determinants of Molecular Initiating Events in Oxide Particle Toxicity. *Int. J. Mol. Sci.* **2023**, *24*, 11482. https://doi.org/10.3390/ijms241411482.

(2) Zhuravlev, L. T. The Surface Chemistry of Amorphous Silica. Zhuravlev Model. *Colloids Surfaces A, Physicochem. Eng. Asp.* **2000**, *173*, 1–38. https://doi.org/10.1016/s0927-7757(00)00556-2.

(3) Di Paola, A.; Bellardita, M.; Palmisano, L.; Barbieriková, Z.; Brezová, V. Influence of Crystallinity and OH Surface Density on the Photocatalytic Activity of TiO₂ Powders. *J. Photochem. Photobiol. A Chem.* **2014**, *273*, 59–67. https://doi.org/10.1016/j.jphotochem.2013.09.008.

(4) Wu, C. Y.; Tu, K. J.; Deng, J. P.; Lo, Y. S.; Wu, C. H. Markedly Enhanced Surface Hydroxyl Groups of TiO₂ Nanoparticles with Superior Water-Dispersibility for Photocatalysis. *Materials,* **2017**, *10* (5), 566. https://doi.org/10.3390/ma10050566.

(5) Wendlandt, W. W. Thermogravimetric and Differential Thermal Analysis of (Ethylenedinitrilo)Tetraacetic Acid and Its Derivatives. *Anal. Chem.* **1960**, *32* (7), 848–849. https://doi.org/10.1021/ac60163a604.

(6) Stolzenburg, P.; Garnweitner, G. Experimental and Numerical Insights into the Formation of Zirconia Nanoparticles: A Population Balance Model for the Nonaqueous Synthesis. *React. Chem. Eng.* **2017**, *2* (3), 337–348. https://doi.org/10.1039/c7re00005g.

(7) Gengenbach, T. R.; Major, G. H.; Linford, M. R.; Easton, C. D. Practical Guides for X-Ray Photoelectron Spectroscopy (XPS): Interpreting the Carbon 1s Spectrum. *J. Vac. Sci. Technol. A Vacuum, Surfaces, Film.* **2021**, *39* (1). https://doi.org/10.1116/6.0000682.

(8) Cristoforetti, G.; Pitzalis, E.; Spiniello, R.; Ishak, R.; Giammanco, F.; Muniz-Miranda, M.; Caporali, S. Physico-Chemical Properties of Pd Nanoparticles Produced by Pulsed Laser Ablation in Different Organic Solvents. *Appl. Surf. Sci.* **2012**, *258* (7), 3289–3297. https://doi.org/10.1016/j.apsusc.2011.11.084.
